# High-Rate, Selective Electrosynthesis of Cyclohexanone Oxime via In Situ Generation and Release of Hydroxylamine on Bismuth

**DOI:** 10.1021/jacs.6c05163

**Published:** 2026-05-29

**Authors:** Lei Shi, Shuyi Cao, Libang Xu, Parker Ballard-Kyle, Yuanqi Liu, Wenjin Sun, Hua Zhou, Sen Zhang, Hongliang Xin, Huiyuan Zhu

**Affiliations:** † Department of Chemistry, 2358 University of Virginia, Charlottesville, Virginia 22904, United States; ‡ Department of Chemical Engineering, University of Virginia, Charlottesville, Virginia 22903, United States; § Department of Chemical Engineering, Virginia Polytechnic Institute and State University, Blacksburg, Virginia 24060, United States; ∥ X-ray Science Division, Advanced Photon Source, 1291 Argonne National Laboratory, Lemont, Illinois 60439, United States

## Abstract

Oxime compounds are key industrial intermediates for nylon precursors and commodity chemicals. However, conventional routes rely on multistep reactions and hydroxylamine (NH_2_OH) salts, raising significant safety and sustainability concerns. Although electrosynthesis offers an alternative, oxime formation on *d*-block transition metals suffers from poor selectivity, as nitrogen oxyanion intermediates bind strongly to the surface and are readily over-reduced to ammonia. Here, we report morphology-controlled *p*-block bismuth rhombic dodecahedra (Bi RDs) that promote *in situ* NH_2_OH generation and its desorption into the electrolyte, enabling an electrochemical-chemical decoupled route for cyclohexanone oxime (CHO) synthesis. Bi RDs deliver nearly 100% Faradaic efficiency (FE) at −0.5 V vs. RHE and a yield of 1.4 mmol h^–1^ cm^–2^ at −0.9 V vs. RHE in an H-cell, while maintaining a CHO selectivity of nearly 100% at 100 mA cm^–2^ in a flow cell. Under identical conditions, *d*-block electrodes (Cu, Pd, Ag) show FE below 30%. Density functional theory calculations reveal that Bi 6*p* orbital-derived surface states weaken intermediate binding and facilitate NH_2_OH desorption, suppressing over-reduction. Kinetic analysis, post-addition trapping experiments, and *in situ* ATR-FTIR and Raman spectroscopy suggest the following reaction mechanism: NH_2_OH is selectively generated at the electrode surface, released as a freely diffusing intermediate, and undergoes homogeneous condensation with cyclohexanone in the bulk electrolyte, bypassing the surface-confined Langmuir–Hinshelwood pathway. These findings demonstrate that regulating intermediate desorption through *p*-block orbital chemistry provides a general strategy for achieving high selectivity in electro-organic nitrogen synthesis.

## Graphical abstract

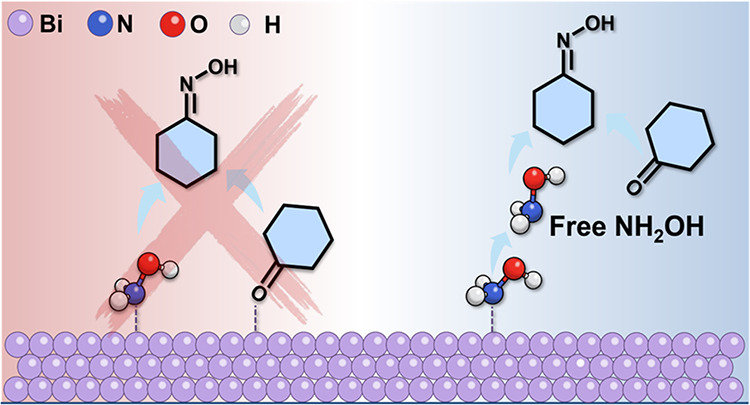

## Introduction

The electrification of chemical manufacturing processes, together with the urgent need to mitigate anthropogenic disruption of the nitrogen cycle, has fueled growing interest in the direct conversion of inorganic nitrogen oxyanions (NO_*x*_^–^, e.g., NO_3_^–^ and NO_2_^–^) into high-value organic nitrogen compounds.^1–3^ Among these target products, oximes are particularly attractive as they are important building blocks in industrial chemical synthesis and play a pivotal role across polymer value chains.^4,5^ For example, under mild conditions, cyclohexanone oxime (CHO) can be readily converted to ε-caprolactam via the Beckmann rearrangement, a key precursor to nylon-6. Notably, nylon-6 is the most recyclable form of nylon, as it can be depolymerized back to caprolactam, making it highly appealing for circular-economy applications.^6,7^ The large and growing industrial demand for CHO thus creates a compelling motivation to develop a sustainable, electrified synthesis route.

Current industrial CHO production, however, relies on chemistries that are fundamentally at odds with sustainability and safety goals. Traditional industrial oximation typically involves reacting cyclohexanone (CYC) with hydroxylamine (NH_2_OH) salts such as sulfate ((NH_2_OH)_2_·H_2_SO_4_) or phosphate ((NH_2_OH)_3_·H_3_PO_4_). However, these salts are highly reactive and can readily detonate upon heating, necessitating stringent safety measures to control their preparation, storage, transportation, and process operation.^8,9^ In recent years, TS-1 (titanium silicalite-1)-catalyzed ammoximation has emerged as an alternative route, in which NH_2_OH is generated *in situ* from the reaction of ammonia (NH_3_) with hydrogen peroxide and is immediately consumed in the oximation step, thereby reducing reliance on NH_2_OH salts.^10,11^ Nevertheless, this approach introduces the strong oxidant hydrogen peroxide (H_2_O_2_), which poses potential fire and explosion hazards and can undergo vigorous decomposition upon heating or in the presence of contaminants.^12^ These persistent safety and sustainability concerns underscore the need for fundamentally greener routes to CHO. This motivates an electrocatalytic approach that can generate CHO directly from stable, abundant NO_*x*_^–^ feedstocks in wastewater under ambient conditions, bypassing both NH_2_OH salts and H_2_O_2_ altogether.^13,14^

However, realizing this vision necessitates overcoming the fundamental selectivity challenges inherent in the electrochemical reduction of NO_*x*_^–^.^15^ The multielectron and multiproton reduction process of NO_*x*_ produces a series of nitrogen-containing intermediates, the most critical of which is NH_2_OH.^16,17^ These intermediates are highly susceptible to over-reduction and conversion to NH_3_, especially under high current density conditions.^18,19^ In traditional heterogeneous electrocatalytic systems, C–N bond formation follows the classical Langmuir–Hinshelwood mechanism, wherein nitrogen-containing intermediates and carbonyl substrates must be coadsorbed simultaneously within the electrochemical double layer at the electrode–electrolyte interface.^20,21^ This surface-confinement constraint is particularly pronounced for d-block transition metals; strong, d-band-mediated chemisorption excessively stabilizes the *NH_2_OH intermediate, thereby prolonging its residence time on the surface.^22,23^ This renders the subsequent proton-coupled electron transfer process (leading to conversion into NH_3_) more kinetically favorable, while conversely inhibiting its C–N coupling reaction with the sterically hindered CYC molecule.^24,25^ The Langmuir–Hinshelwood framework, together with the d-band-dominated binding mechanism, collectively establishes a fundamental upper limit for the selectivity attainable by d-block heterogeneous catalytic systems. Consequently, recent research advancements have explored a conceptually distinct strategy: allowing NH_2_OH to desorb from the electrode surface and subsequently undergo a homogeneous condensation reaction with CYC within the bulk electrolyte solution, thereby completely circumventing the constraints imposed by surface confinement.^26^ Heterogeneous copper-based catalysts provided the initial proof-of-concept for this NH_2_OH-mediated reaction pathway. Concurrently, molecular electrocatalysts, specifically ZnPc, CoPc and FePc, achieved significantly higher NH_2_OH selectivity through coordination field engineering; notably, FePc facilitated a completely decoupled two-step strategy, involving the initial electrochemical synthesis of free NH_2_OH followed by its utilization in a separate oximation reactor.^27–29^ Furthermore, in systems utilizing NO as the nitrogen source, by modulating the local NO coverage on the surface of Ru-doped Ag catalysts, reaction selectivity was successfully steered toward CHO products at ampere-level current densities.^30^ Despite these advancements, molecular catalyst systems still face inherent challenges, including limited stability under prolonged electrolysis, sensitivity to operating pH, and the need to either physically separate reactors or tightly control the concentration ratio between electrochemically generated NH_2_OH and the carbonyl substrate. For heterogeneous metal catalysts, however, the orbital-level electronic origins governing spontaneous desorption of NH_2_OH have yet to be established; moreover, design principles that correlate crystal facet geometry with the desorption selectivity of intermediates remain lacking.

To overcome these shortcomings, a heterogeneous catalyst is needed whose surface electronic structure is inherently unfavorable for the retention of *NH_2_OH, not through molecular coordination engineering, but through the orbital properties of the metal itself. p-block bismuth (Bi) thus becomes a particularly attractive candidate for this purpose.^31–33^ Unlike d-block transition metals, where d-band-mediated chemisorption is the root cause of *NH_2_OH overstability, Bi possesses oriented 6p orbitals and inert 6s^2^ lone pairs of electrons.^34^ This results in its valence band being filled or nearly half-filled, significantly reducing its oxygen affinity–meaning its binding force with nitrogen-containing intermediates is inherently weaker, and the surface is more inclined to release *NH_2_OH into the bulk electrolyte rather than confining it to the surface for further reduction. This stereoelectronic property positions Bi uniquely, enabling spontaneous desorption of *NH_2_OH within a single-pot electrochemical cell. This approach retains the durability and scalability advantages of heterogeneous systems while circumventing the stability limitations of molecular catalysts and the engineering complexities of physically decoupled two-step reactors.

In this study, we demonstrate that morphology-controlled *p*-block Bi rhombic dodecahedra (RDs) enable highly efficient electrochemical synthesis of CHO, achieving a Faradaic efficiency (FE) of up to 99.2% and a high production rate of 1.4 mmol h^–1^ cm^–2^ in an H-type electrochemical cell, substantially outperforming Bi nanoparticles (NPs) and other electrodes (Cu, Ag, Pd, SnO_*x*_ and In_2_O_3_). Through post-addition trapping experiments, *in situ* attenuated total reflectance fourier transform infrared (ATR-FTIR) spectroscopy, Raman spectroscopy, and kinetic analysis, we established the reaction mechanism for C–N bond formation within this heterogeneous system. Specifically, this process does not follow a surface-confined Langmuir–Hinshelwood pathway; rather, it proceeds via a homogeneous condensation reaction between freely diffusing NH_2_OH and CYC within the bulk electrolyte phase. This decoupled electrochemical–chemical reaction pathway is further supported by density functional theory (DFT) calculations. The free-energy profiles clearly show that, on the Bi RD surface, desorption of the NH_2_OH intermediate is thermodynamically highly favorable across all relevant Bi surfaces, effectively suppressing over-reduction side reactions that are commonly observed in other heterogeneous catalytic systems (e.g., Cu-based catalysts). Collectively, these findings establish a design principle for heterogeneous electro-organic synthesis: p-block orbital chemistry can be exploited to regulate spontaneous intermediate desorption, enabling one-pot electrochemical-chemical coupling with near-unity selectivity and high production rates on a durable, scalable platform.

## Results and Discussion

Inspired by the intrinsically slow hydrogen evolution kinetics and high overpotential of *p*-block metal Bi,^35^ we aim to tailor its local coordination environment through precise control of surface geometry.^36^ This strategy seeks to suppress the competing hydrogen evolution reaction (HER) while promoting the selective generation and release of target intermediates in the NO_2_^–^ reduction system. By constructing nanostructures with well-defined geometric boundaries and synergistic exposure of (104) and (110) facets, the spatially oriented 6p orbital states of Bi can be leveraged to regulate the adsorption–desorption equilibrium of intermediates. This ensures sufficient intermediate concentration for solution-phase pathways. Consequently, we developed a precise colloidal synthesis route and obtained highly uniform Bi RD nanocrystals with the desired crystal plane combinations.

Transmission electron microscopy (TEM) and high-angle annular dark-field scanning transmission electron microscopy (HAADF-STEM) characterization revealed that the Bi RDs possess a highly regular rhombic dodecahedral shape with well-defined edges and vertices (Figure 1a,b) with a size of 36 nm ± 6 nm (Figure S1a), indicating that the synthesis system effectively suppressed disordered growth and achieved precise control over the nucleation and crystallization processes. For comparison, spherical Bi nanoparticles with a similar size to Bi RDs (Figure S1b) were also synthesized. This thermodynamically stable but less well-defined surface served as an ideal control for evaluating the relationship between morphology, crystal facet distribution, and catalytic performance. Further high-resolution (HR-) TEM and fast Fourier transform (FFT) analysis confirmed the single-crystallinity of the Bi RDs (Figure 1c). Multiregion lattice analysis of Bi RDs revealed characteristic spacings of 0.335, 0.237, and 0.227 nm, corresponding to the (012), (104), and (110) planes of rhombohedral Bi (space group: R3̅m ), respectively (Figure S2). In contrast, the HRTEM images of Bi NPs primarily exhibited lattice fringes corresponding to the thermodynamically most stable (012) plane (Figures 1d–f and S3). This confirms that the specific polyhedral shape of Bi RDs enables the coexposure of multiple low-index facets, offering a tailored surface structure distinct from that of the isotropic Bi NPs. Based on stereoelectronic considerations,^37^ the coexistence of multiple crystal facets may alter the local geometry of surface Bi atoms and the distribution of *p*-orbital-related surface states, thereby affecting the surface residence and desorption tendency of nitrogen-containing intermediates (especially NH_2_OH-related species). The morphology and elemental distribution were further assessed by HAADF-STEM and energy-dispersive X-ray spectroscopy (EDS) mapping (Figure 1g). EDS mapping shows a homogeneous Bi distribution across the structure, with no compositional segregation. A weak oxygen signal is also detected, mainly at the particle periphery, consistent with the formation of a thin native oxide layer upon air exposure, a phenomenon commonly observed in metallic Bi materials.^38^

**Figure 1:**
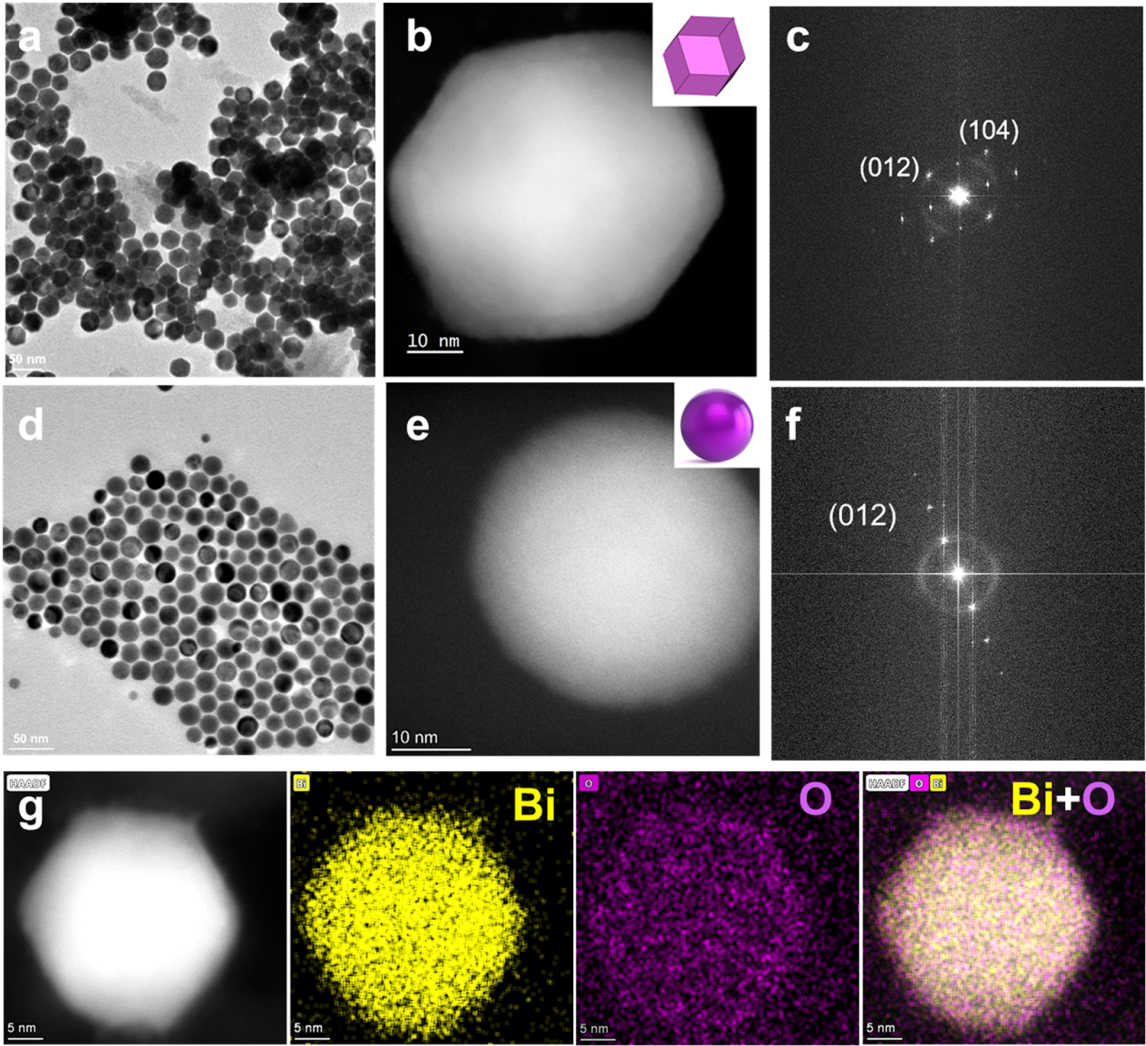
Electron microscopic characterization of as-synthesized Bi. (a) TEM image of Bi RDs. (b) HAADF-STEM of Bi RDs. (c) Corresponding FFT pattern of the Bi RDs. (d) TEM image of Bi NPs. (e) HAADF-STEM of Bi NPs. (f) Corresponding FFT pattern of the Bi NPs. (g) HAADF-STEM image and corresponding elemental mapping of Bi RDs.

The crystallinity of the catalysts was evaluated by X-ray diffraction (XRD). As shown in Figure 2a, both Bi RDs and Bi NPs exhibited diffraction peaks consistent with the rhombohedral Bi phase (PDF No. 44–1246). Notably, the relative intensities of the (104) and (110) diffraction peaks are significantly enhanced in Bi RDs relative to the main (012) peak compared with Bi NPs. This intensity difference is consistent with shape-induced preferential orientation and suggests an increased contribution of the (104) and (110) facets in the rhombic dodecahedral morphology.^39^ X-ray photoelectron spectroscopy (XPS) confirmed the chemical composition of the sample, with the survey spectrum displaying characteristic Bi 4f, 4p, and 5d peaks (Figure S4). In the high-resolution Bi 4f spectrum (Figure 2b), the dominant peaks at ∼157.0 and ∼162.3 eV are assigned to metallic Bi^0^. A high-binding-energy component corresponding to Bi^3+^ is also observed, which is attributed to the presence of a thin native oxide layer formed upon air exposure. The synthesized Bi NPs exhibit similar XPS characteristics (Figure S5).

**Figure 2:**
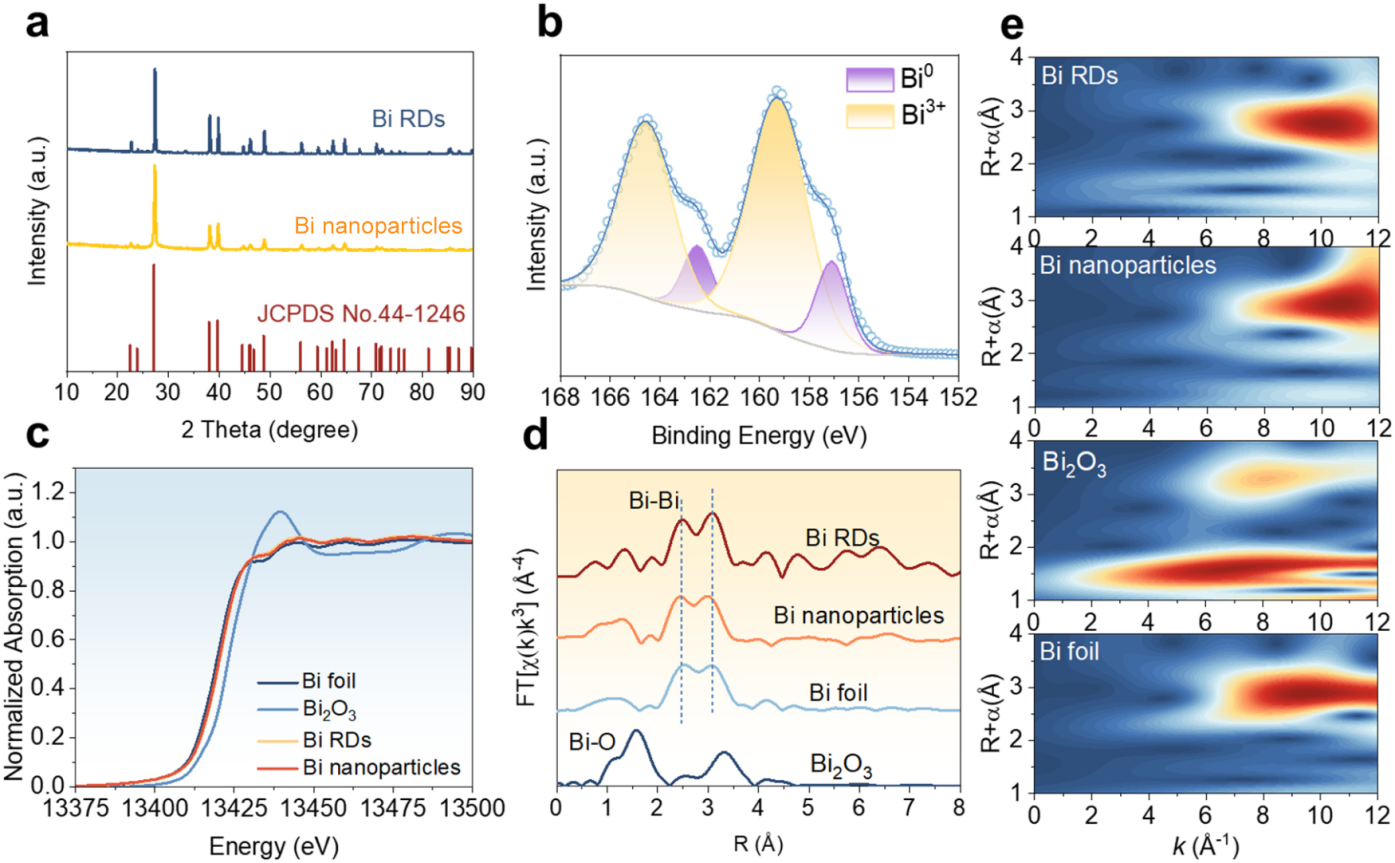
Structural characterization of as-synthesized Bi. (a) XRD patterns of Bi RDs and Bi NPs. (b) High-resolution spectrum of Bi 4f electrons of Bi RDs. (c) Bi L_3_-edge XANES spectra of Bi RDs, Bi NPs and standard reference samples. (d) Bi R-space EXAFS spectra of Bi RDs, Bi NPs and standard reference samples. (e) Wavelet transform map of the Bi L_3_-edge EXAFS signals of Bi RDs, Bi NPs and standard reference samples.

To further probe the local coordination and valence states, synchrotron radiation-based X-ray absorption spectroscopy (XAS) was conducted. The Bi L_3_-edge X-ray absorption near edge structure (XANES) spectra of Bi RDs and Bi NPs (Figure 2c) closely match that of Bi foil, further confirming the predominantly metallic nature of both samples. In the R-space extended X-ray absorption fine structure (EXAFS) spectra (Figure 2d), the prominent features at approximately 2.5 and 3.0 Å (without phase correction) are attributed to Bi–Bi scattering paths. This assignment is further supported by the wavelet transform (WT) plot (Figure 2e), which exhibits a maximum intensity at approximately 12 Å^–1^, characteristic of Bi–Bi coordination. Quantitative fitting of the EXAFS data (Figures S6, S7 and Table S1) confirmed that the coordination number is consistent with an ordered metallic lattice. Raman spectroscopy (Figure S8) further confirmed the metallic lattice, with the characteristic A_1g_ mode observed at ∼93 cm^–1^. Notably, the A_1g_ peak intensity is significantly higher for Bi RDs than for Bi NPs, likely due to differences in particle size and crystallinity, as this mode is highly sensitive to coherent domain size and lattice order.^40^ The enhanced A_1_g intensity in Bi RDs suggests higher crystallinity and larger effective grain size, whereas Bi NPs likely contain more surface defects or disordered regions.

We first evaluated the electrocatalytic activity of various metal electrodes (Bi, Cu, Ag, Pd) for CHO synthesis in a standard H-type electrolytic cell. Linear sweep voltammetry (LSV) curves revealed distinct polarization behaviors across the metals. As shown in Figures S9 and S10, *d*-block transition metals such as Cu exhibit significantly higher cathodic current densities, whereas Bi-based catalysts show relatively mild current responses. We then recorded the LSV curves on Bi in different electrolyte environments (Figure 3a). The results showed that the current density was minimal in pure 0.5 M KOH solution but increased significantly upon adding 0.5 M KNO_2_, demonstrating that Bi RDs exhibit strong catalytic activity for nitrite reduction. Further addition of the CYC substrate induced an additional shift in the polarization curve, consistent with rapid consumption of interface-generated active intermediates by the organic substrate. Importantly, the lower total current observed on Bi does not indicate lower activity; rather, it reflects its excellent suppression of the HER, and crucially, prevents over-reduction of nitrogen species to NH_3_.

**Figure 3:**
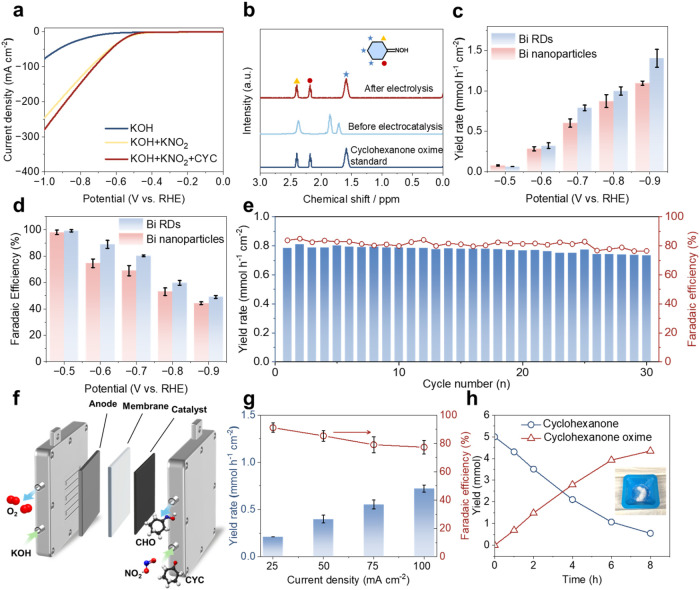
Performance evaluation of electrocatalytic CHO synthesis. (a) LSV curves of Bi RDs in different electrolytes. (b) ^1^H NMR of electrolyte before and after Bi RD catalyzed reaction and standard CHO samples (δ ≈ 2.48, 2.20, 1.59 ppm). (c) CHO yield rate on Bi RDs and Bi NPs at different applied potentials. (d) FE of CHO on Bi RDs and Bi NPs at different applied potentials. (e) CHO yield rate and corresponding FE on Bi RDs at −0.7 V (vs RHE) for 30 consecutive cycles. (f) Reaction setup (flow cell) for the electrosynthesis of CHO, the flow rate is 20 mL min^–1^. (g) CHO yield rate and corresponding FE on Bi RDs at different current density in flow cell measurements. (h) Time-dependent CYC conversion and CHO yield in 100 mL electrolyte in flow cell (The inset shows a photograph of the CHO product after 8 h of electrolysis).

The liquid-phase products after electrolysis were analyzed qualitatively and quantitatively by ^1^H NMR and UV–vis (Figures S11 and S12). Figure 3b compares the ^1^H NMR spectrum of the Bi RDs-catalyzed electrolyte with pure CYC and CHO standards. The observed resonance peaks at ∼2.48, ∼2.20 and ∼1.59 ppm match those of the CHO standard, with no other significant organic byproduct detected, confirming the highly selective formation of the target product, CHO. Further quantitative analysis revealed a pronounced selectivity gap between *d-*block and some other *p*-block catalysts (SnO_*x*_ and In_2_O_3_) (Figures 3c,d, and S13–S15). Conventional *d*-block transition metals (Pd, Cu, Ag) suffer from severe over-reduction to NH_3_, resulting in CHO FEs typically below 30%. Although SnO_*x*_ and In_2_O_3_, both p-block catalysts, also exhibit measurable CHO selectivity, their performance is still inferior to Bi RDs. This observation provides preliminary evidence for establishing a general design principle for p-block metal catalysts. This trend is consistent with the characteristic that nitrogen intermediates generally have weaker binding forces on p-block metal surfaces. On such surfaces, the tendency for NH_2_OH to be over-reduced to NH_3_ is reduced due to the lack of partially filled d-band states. In contrast, Bi RDs exhibited superior selectivity, achieving a CHO FE of ∼99.2% at −0.5 V (vs reversible hydrogen electrode, RHE), nearly 100% selectivity, and a yield rate of 1.4 mmol h^–1^ cm^–2^ at −0.9 V (Figures S16, S17 and Table S2). Compared to other transition metals, at a mild cathodic potential (−0.5 V vs RHE), the Faradaic efficiency for NH_3_ on Bi RDs remains below 5%; however, as the potential shifts negatively to −0.7 and −0.8 V vs RHE, the NH_3_ Faradaic efficiency increases to approximately 20%, consistent with the trend of enhanced proton-coupled electron transfer processes at higher overpotentials. These results strongly support our mechanistic hypothesis: the unique electronic structure of the Bi-based catalyst makes the interfacial formation of the NH_2_OH intermediate energetically favorable for desorption from the surface, rather than remaining on the surface to be further reduced to NH_3_ (Figure S18). Furthermore, morphological engineering substantially improved performance. While Bi NPs also showed high selectivity (∼98%), Bi RDs achieved a significantly higher yield at the same potential. To decouple surface area effects from intrinsic activity, the electrochemical active surface area (ECSA) was measured using double-layer capacitance (Cdl) (Figures S19 and S20). After normalization, the partial current density of CHO on Bi RDs was significantly higher than that of Bi NPs (Figure S21), suggesting that the distinct surface structure of Bi RDs contributes to its higher intrinsic activity.

Bi RDs also demonstrated outstanding structural stability and broad electrolyte compatibility, maintaining high selectivity in alkaline, phosphate buffer saline, and potassium bicarbonate solutions (Figures S22 and S23). Long-term stability tests showed negligible decay in FE or yield rate after 30 electrolysis cycles (Figure 3e), and postreaction XRD and TEM characterization confirmed the structural stability (Figure S24). We further integrated Bi RDs into a gas diffusion layer (GDL)-based flow cell to assess their potential for scalable implementation (Figure 3f). The cell with the Bi RD catalyst operated stably for 8 h at a high current density of 100 mA cm^–2^, maintaining a cell voltage of ∼2.6 V, and a FE above 80% (Figures 3g,h, S25, S26). Following 8 h of electrolysis, the recovery rates for both carbon and nitrogen balance data exceeded 95%, further confirming that no significant loss of carbon or nitrogen occurred within the system and thereby demonstrating its stability (Figure S27). Sustained high selectivity and FE at industrial current densities demonstrate the feasibility of the intermediate release electrosynthesis strategy.

To probe the bond formation in the oximation reaction, we analyzed the reaction coordinate and the transient behavior of intermediates at the electrode–electrolyte interface. We hypothesize that the catalytic process on the Bi RDs surface follows an electrochemical–chemical spatial decoupling pathway (Figure 4a): the Bi surface selectively reduces nitrite to release NH_2_OH intermediates, while the subsequent C–N coupling occurs via homogeneous condensation in the bulk solution. To verify this proposed mechanism, we performed control experiments using electrochemistry and ^1^H NMR (Figure S28). In the absence of CYC substrate, no CHO peaks were detected in the Bi-catalyzed electrolyte. Upon the addition of CYC to the pre-electrolyzed solution, characteristic CHO peaks appeared. In contrast, the Cu NP system showed no CHO peaks under the same conditions, indicating that on *d*-block metal surfaces, the reaction intermediate remains tightly bound and is rapidly over-reduced to NH_3_. To quantitatively assess the spatial decoupling of the electrochemical and chemical coupling steps, we examined the concentration effect of CYC substrate on reaction performance (Figure 4b). As the CYC concentration increased from 0.01 to 0.1 M, the CHO FE rose from 50.2 to 80.3%, while the yield increased linearly from 0.47 to 0.78 mmol h^–1^ cm^–2^, suggesting a homogeneous condensation process in which CYC in solution scavenges the *in situ*-generated NH_2_OH. A control experiment (Table S3) also clearly showed that NH_2_OH and CYC spontaneously undergo efficient condensation to produce CHO even without applied potential, confirming the coupling is a chemical step.

**Figure 4:**
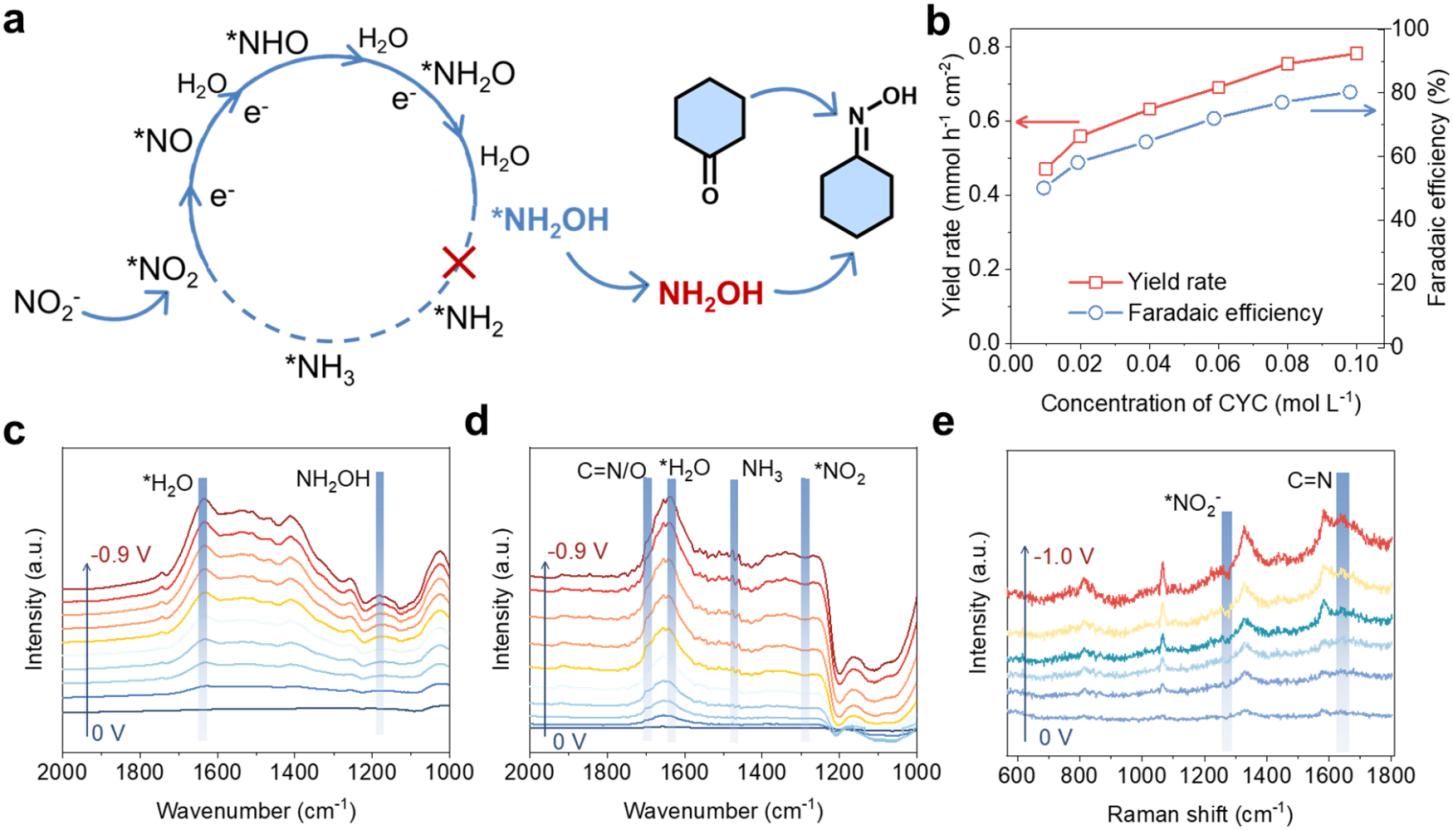
Mechanism investigations and *in situ* characterization of CHO electrosynthesis. (a) Schematic illustration of the synthesis pathway of CHO on the catalyst. (b) Effect of CYC substrate concentration on electrocatalytic performance at −0.7 V vs RHE. (c) *In situ* ATR-FTIR spectra of Bi RDs in the electrolyte containing 0.5 M KOH + 0.5 M KNO_2_. (d) *In situ* ATR-FTIR spectra of Bi RDs in the electrolyte containing 0.5 M KOH + 0.5 M KNO_2_ + 0.1 M CYC. (e) Potential-dependent *in situ* electrochemical Raman spectra for oxime electrosynthesis.

To monitor the transient evolution of intermediates at the electrode–electrolyte interface, we combined *in situ* attenuated total reflection Fourier transform infrared spectroscopy (ATR-FTIR) with electrochemical Raman spectroscopy. In the electrolyte containing only nitrite (Figure 4c), as the applied potential was shifted cathodically, an absorption band at ∼1180 cm^–1^ gradually intensified, which is assigned to the deformation vibration of the *NH_2_OH intermediate.^41^ Crucially, this characteristic peak exhibited no appreciable potential-dependent frequency shift (Stark shift), indicating that the generated NH_2_OH was not strongly adsorbed on the electrode surface but instead existed as a freely diffusing species within the diffusion layer near the interface (Figure S29). Upon introducing the CYC substrate (Figure 4d), the infrared spectrum evolved markedly, with a new characteristic band emerging near ∼1700 cm^–1^, attributed to the C=N stretching vibration in the oxime product.^42^ Notably, this key signal corresponding to CHO formation was absent in Figure 4c (without the substrate), confirming that C=N bond formation depends on the substrate scavenging the intermediates released at the interface. *In situ* electrochemical Raman spectroscopy further supported this finding (Figure 4e), where the characteristic oxime vibrational fingerprint near 1650 cm^–1^ emerges upon potential scanning under reducing conditions, confirming the interfacial formation dynamics of the target product.

Density functional theory (DFT) calculations were performed to investigate the reaction pathways of nitrite reduction on metal surfaces. Previous studies have proposed multiple pathways toward NH_3_ formation depending on electrolyte pH and catalyst identity;^43,44^ however, there remains no consensus on the critical intermediates or the governing reaction factors. Figure S30 presents free energy diagrams of representative reaction pathways on Bi surfaces at −0.5 V vs RHE under alkaline conditions. Among the pathways considered, we focus on *NO_2_ → *NO → *NHO → *NH_2_O → *NH_2_OH → *NH_2_ → NH_3_, as it is thermodynamically the most favorable route across all investigated Bi surfaces. Figure 5 presents schematic reaction intermediates together with the grand-canonical DFT free energy profiles for NO_2_RR to NH_3_ on Bi, Ag, Cu, and Pd at −0.5 V vs RHE, and shows the corresponding surface slab models (the Ag slab is shown as a representative for Ag, Cu, and Pd); detailed optimized intermediates on Bi are provided in Figure S31. Under alkaline conditions, NO_2_RR is assumed to proceed through a sequence of deoxygenation steps leading to *NO formation, followed by further hydrogenation toward NH_3_. This process is likely mediated by *NHO and involves adsorbed NH_2_OH as a key intermediate. While *NH_2_OH is generally regarded as a transient species that is rapidly reduced to NH_3_ on many metal catalysts,^45,46^ our calculations indicate a distinct behavior on Bi surfaces. Specifically, *NH_2_OH exhibits a comparatively more favorable desorption free energy on Bi, making its surface binding less favorable than on Ag, Cu and Pd surfaces (Figure 5a). Among the three Bi facets, Bi(110) exhibits the most negative NH_2_OH desorption free energy (−1.30 eV), compared with Bi(012) (−1.16 eV) and Bi(104) (−1.14 eV), highlighting the facet dependence of NH_2_OH desorption on Bi surfaces. Here, a more negative desorption free energy corresponds to more favorable desorption, meaning that NH_2_OH is more likely to desorb from the Bi(110) surface. Consequently, NH_2_OH is more likely to directly desorb from the Bi surface into the electrolyte before further reduction steps. This tendency for NH_2_OH desorption increases its availability in the solution phase, thereby facilitating subsequent homogeneous or interfacial C–N coupling reactions. In contrast, stronger *NH_2_OH adsorption on Ag, Cu and Pd stabilizes this intermediate on the surface, favoring continued reduction to NH_3_ and suppressing its release into the electrolyte. Notably, similar trends in *NH_2_OH adsorption energetics and desorption propensity on Bi are also observed at a more negative potential of −0.9 V vs RHE (Figure S32), indicating that this behavior is robust across a wide electrochemical potential window rather than being specific to a single operating condition. Because NH_2_OH binds too weakly to produce clear density of states (DOS) features, NO_2_ adsorption was used instead to probe the orbital characteristics of surface bonding. The O *p* states refer to the summed 2*p*-projected DOS of the two O atoms in adsorbed NO_2_. The projected DOS (PDOS) results suggest that adsorption on Bi, as exemplified by Bi(104), is mainly associated with Bi *p*-state participation, whereas on Ag, Cu, and Pd, the dominant overlap is found in the metal *d*-state region, indicating d-orbital-mediated bonding (Figure 5b). The corresponding N p projected DOS is provided in Figure S33 for completeness.

**Figure 5:**
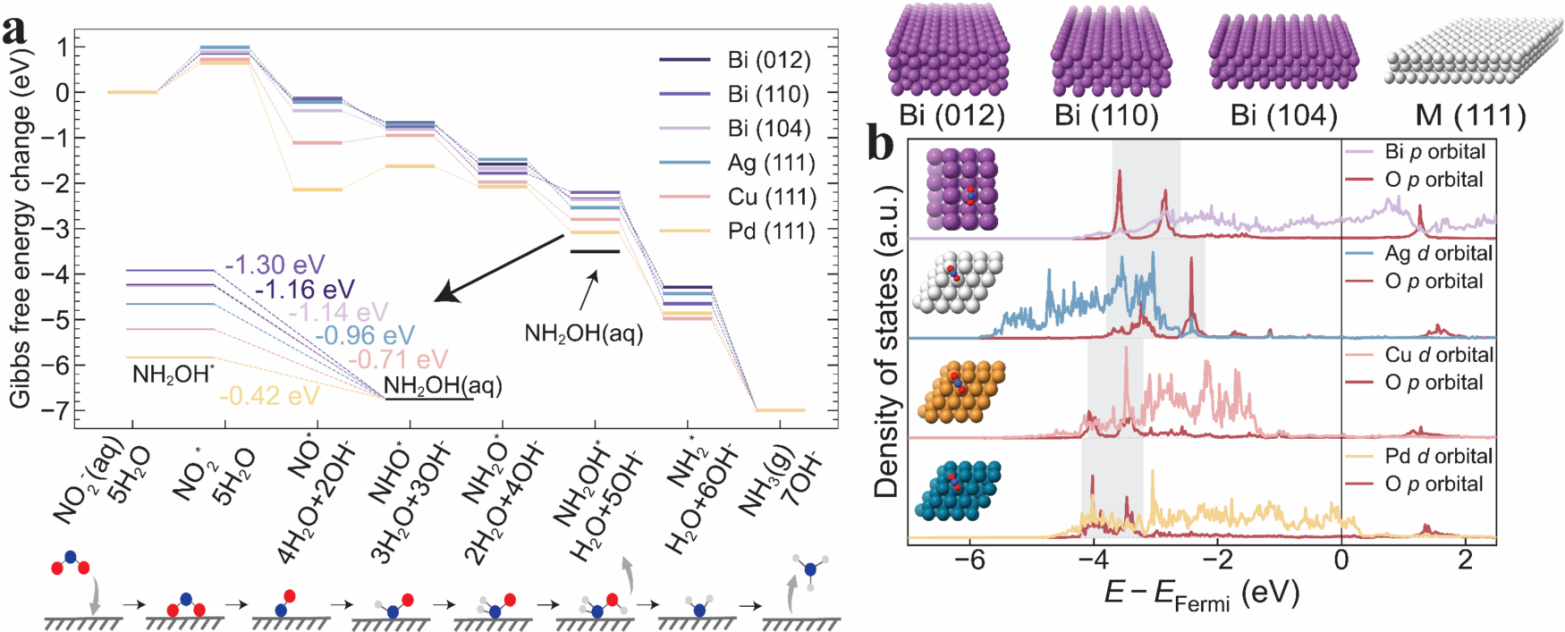
Reaction pathways of NO_2_RR to NH_3_ and PDOS analysis of NO_2_ adsorption. (a) Grand canonical DFT-calculated free energy profiles for the electrochemical reduction of NO_2_^–^ to NH_3_ at −0.5 V vs RHE on Bi (012), Bi (110), Bi (104), Ag (111), Cu (111), and Pd (111) surfaces. Gibbs free energy changes for each elementary proton–electron transfer step are shown relative to NO_2_^–^(aq). The free formation energies of the desorbed intermediate NH_2_OH (aq) are explicitly indicated. Schematic illustrations of surface-bound intermediates along the reaction pathway are included for clarity. (b) PDOS of adsorbed NO_2_ on the corresponding surfaces. The shaded regions mark the main overlap between Bi *p* and O *p* states on Bi and between metal *d* and O *p* states on Ag, Cu, and Pd.

## Conclusion

In summary, we demonstrate that morphology-controlled *p*-block Bi RDs enable highly efficient and selective electrocatalytic CHO synthesis, reaching a FE of 99.2% and surpassing the yield limitations of conventional *d*-block catalysts. *In situ* spectroscopy and kinetic analysis show that the Bi surface generates and releases NH_2_OH, enabling C–N bond formation via homogeneous condensation in the bulk electrolyte, establishing a NH_2_OH-mediated, spatially decoupled pathway. The exceptional selectivity stems from the unique stereoelectronic effect, through which the orientation of Bi’s 6*p* orbitals modulate adsorption–desorption, allowing *NH_2_OH to desorb before forming NH_3_ and thereby circumventing the over-reduction pathway. By managing intermediate flux through spatially decoupled electrochemical and chemical steps, this strategy both valorizes NO_*x*_ waste and guides the design of more efficient electrosynthesis systems leveraging *p*-block metal orbitals.

## Supplementary Material



## Data Availability

All relevant data supporting the findings of this study are available in the article and Supporting Information. Source data are provided with this paper.

## Notes

Experimental procedures, additional characterization data, electrochemical measurements, in situ spectroscopic data, and computational details (PDF)
