# Supplementary material for: High-Rate, Selective Electrosynthesis of Cyclohexanone Oxime via In Situ Generation and Release of Hydroxylamine on Bismuth
Source: J Am Chem Soc. 2026 May 29;148(22):23109–17. doi: 10.1021/jacs.6c05163 (PMC13266689; doi:10.1021/jacs.6c05163)
Supplement: Supplementary file 1 [file ja6c05163_si_001.pdf]

Supporting Information for

High-Rate, Selective Electrosynthesis of Cyclohexanone Oxime via In Situ Generation and Release of Hydroxylamine on Bismuth

Lei Shi[1#], Shuyi Cao[3#], Libang Xu[1], Parker Ballard-Kyle[1], Yuanqi Liu[2], Wenjin Sun[1], Hua Zhou[4], Sen Zhang[1], Hongliang Xin[3], Huiyuan Zhu[1, 2*]*

[1] Department of Chemistry, University of Virginia, Charlottesville, Virginia 22904, USA.

[2] Department of Chemical Engineering, University of Virginia, Charlottesville, Virginia 22903, USA

[3] Department of Chemical Engineering, Virginia Polytechnic Institute and State University, Blacksburg, Virginia 24060, USA.

[4] X-ray Science Division, Advanced Photon Source, Argonne National Laboratory, Lemont, Illinois 60439, USA

[#]These authors contributed equally to this work.

Email: kkx8js@virginia.edu; hxin@vt.edu

Experimental Section

1. Materials:

Bismuth neodecanoate, bismuth acetate ($Bi(OAc)_3$), bismuth nitrate pentahydrate ($Bi(NO_3)_3$), 1-octadecene (ODE, 90%), 1-dodecanethiol (DDT, 98%), trioctylphosphine (TOP, 98%), potassium hydroxide (KOH), potassium nitrite (KNO_2), copper acetylacetonate ($Cu(acac)_2$), palladium acetylacetonate ($Pd(acac)_2$), silver nitrate ($AgNO_3$), sodium hypochlorite solution (NaClO, 10%), sodium citrate ($C_6H_5Na_3O_7$), salicylic acid ($C_7H_6O_3$), sodium nitroprusside ($Na_2[Fe(CN)_5NO]\cdot 2H_2O$), hydrogen peroxide ($H_2O_2$, 40%), sulfuric acid ($H_2SO_4$, 98%), cyclohexanone (CYC, 99%), cyclohexanone oxime (CHO, 99%), oleylamine (OAm), oleic acid (OAc), 3-(Trimethylsilyl)propionic-2,2,3,3-d_4 acid sodium salt (TMSPA-d_4-Na), sodium hydroxide (NaOH), tin acetate, indium acetate, sodium citrate, and isopropanol were all purchased from Sigma-Aldrich. Hexane and ethanol were technical grade and used as received without further purification.

2. Synthesis of catalysts:

Synthesis of bismuth rhombic dodecahedra (Bi RDs): 1 mmol of bismuth neodecanoate was mixed with 10 mL of ODE and heated to 120°C under a gentle nitrogen flow for 1 hour to remove dissolved water and oxygen. The system was then allowed to cool naturally to 80°C, while 0.24 mL of DDT was injected into the solution to form a bismuth intermediate complex. After 5 minutes, 1.5 mL of TOP was added to the solution, and the system was further cooled to 65°C and maintained at this temperature for 30 minutes. After cooling to room temperature, 25 mL of ethanol was added, and the Bi RDs were collected and washed three times by centrifugation (9000 rpm, 8 minutes).

Synthesis of bismuth nanoparticles (Bi NPs): 1 mmol of $Bi(NO_3)_3$ was dissolved in 10 mL DDT and kept under a gentle nitrogen flow for 1 hour to remove dissolved oxygen. The system was then heated to 178°C at a rate of 10°C min^{-1} and maintained at this temperature for

1 minute. After cooling to room temperature, 25 mL of ethanol was added, and the Bi NPs were collected and washed three times by centrifugation (5000 rpm, 5 minutes)[1].

Synthesis of copper nanoparticles: Copper nanoparticles (Cu NPs) were prepared using a modified method reported in the literature[2]. 0.382 mmol of $Cu(acac)_2$ and 10 mL of OAm were added to a 50 mL three-necked flask. The mixture was heated to 80°C and purged with gentle nitrogen flow for 30 minutes to remove dissolved water and oxygen. The solution was then heated to 230°C at a rate of 10°C min^{-1} and maintained under a nitrogen atmosphere for 3 hours. After the solution cooled to room temperature, it was washed with ethanol and centrifuged at 10000 rpm for 10 minutes. This washing and centrifugation process was repeated three times, after which the product was dispersed in hexane.

Synthesis of palladium nanoparticles: Palladium nanoparticles (Pd NPs) were prepared using a modified method reported in the literature[3]. 0.1523 g of $Pd(acac)_2$, 1.5 ml of TOP, 1.68 ml of OAm, and 1.61 ml of OAc were dissolved in 20 ml of ODE. After degassing with nitrogen at 80°C for 30 minutes, the mixture was heated to 280°C at a heating rate of 20°C min^{-1} and maintained at this temperature for 15 minutes. After the mixed solution cooled to room temperature, it was washed with ethanol and centrifuged at 7000 rpm for 8 minutes. The nanoparticles were washed twice with ethanol and then redispersed in 10 ml of hexane for subsequent use.

Synthesis of silver nanoparticles: Silver nanoparticles (Ag NPs) were prepared using a modified method reported in the literature[4]. 170 mg of $AgNO_3$ was dissolved in 20 ml of OAm. The mixture was heated to 80°C and purged with N_2 for 30 minutes to remove dissolved oxygen. Subsequently, under a nitrogen atmosphere, the mixture was heated to 180°C at a rate of 5°C min^{-1} and maintained at this temperature for 20 minutes. After cooling the mixture to room temperature, the Ag NPs were collected by centrifugation at 9000 rpm for 5 minutes using

ethanol. The nanoparticles were washed twice with ethanol and then redispersed in 10 ml of hexane for further use.

Synthesis of tin oxide (SnO_x): In a typical experimental procedure, 0.1 mmol of Sn acetate, 10 mL of OAm, and 0.5 mL of TOP were added to a 50 mL three-neck flask and allowed to react under stirring. Subsequently, the mixture was heated to 80°C under a nitrogen atmosphere and maintained at this temperature for 30 minutes. Next, the mixture was heated to 200°C at a heating rate of 10°C min^{-1} and held at this temperature for 30 minutes, ultimately yielding a gray solution. After cooling to room temperature, the resulting gray precipitate was isolated by centrifugation, washed repeatedly three times with excess ethanol, and finally redispersed in hexane.

Synthesis of indium oxide (In_2O_3): In a typical reaction, 1.5 mmol of indium acetate and 5 mL of OAm were mixed in a three-necked round-bottom flask, and subsequently maintained at 80°C for 30 minutes under a nitrogen atmosphere. The mixture was then heated to 240°C and held at this temperature for 30 minutes. After the reaction mixture had cooled to room temperature, In_2O_3 nanoparticles were isolated by centrifugation at 5000 rpm for 5 minutes. The resulting yellowish-white solid product was washed with ethanol and then dispersed in hexane.

3. Preparation of carbon-supported catalysts:

A hexane dispersion containing 10 mg of catalyst was mixed with 40 mg of activated carbon (Vulcan XC-72R) and sonicated for 2 hours. The catalyst was first collected by centrifugation. To remove the surface-capping ligands and expose the intrinsic active sites, the collected powder was redispersed in a hydrazine hydrate/ethanol mixed solution and stirred overnight at room temperature. Finally, the catalyst was recovered by centrifugation, washed three times with excess ethanol, and subsequently dried in a vacuum oven.

4. Characterization:

Powder X-ray diffraction (PXRD) patterns of the samples were collected using a Malvern Panalytical Empyrean X-ray diffractometer (Cu Kα radiation, λ = 1.5406 Å). The size and morphology of the samples were characterized using a 120 kV Thermo Fisher Scientific Tecnai Spirit transmission electron microscope (TEM). Bright-field transmission electron microscopy (BF-TEM), high-angle annular dark-field scanning transmission electron microscopy (HAADF-STEM), and energy-dispersive X-ray spectroscopy (EDS) analyses were performed using a Thermo Fisher Scientific Talos F200X TEM equipped with a Super-X EDS detector. The metal content in the catalysts was determined by inductively coupled plasma atomic emission spectrometry using a SPECTRO GENESIS inductively coupled plasma emission spectrometer. The NH_4^+ concentration was quantitatively determined using a UV-Vis spectrophotometer (Agilent 3500) combined with the indophenol blue spectrophotometric method. Gaseous products were quantitatively analyzed using a gas chromatograph (Agilent 7890B). Products were quantitatively analyzed using a nuclear magnetic resonance (NMR) Bruker Neo Nanobay 400. X-ray absorption fine structure (XAFS) spectroscopy (including X-ray absorption near-edge structure, XANES, and extended X-ray absorption fine structure, EXAFS) was performed on the samples at the Advanced Photon Source (APS) (Beamline 12-BM) at Argonne National Laboratory, focusing on the bismuth L_3 edge. Data were collected in fluorescence/transmission mode and processed using the Athena and Artemis programs within the Demeter software package.

5. Electrochemical measurements:

All electrochemical investigations were conducted using a BioLogic electrochemical workstation in a standard three-electrode configuration. Measurements were performed at room temperature in a sealed H-type cell, with the cathodic and anodic compartments separated by a Nafion 117 cation-exchange membrane. The membrane was pre-treated sequentially in 5% H_2O_2 at 80°C for 1 h and 0.5 M H_2SO_4 at 80°C for 1 h, followed by thorough rinsing with

deionized (DI) water. A platinum foil and an Ag/AgCl (3.5 M KCl) electrode served as the counter and reference electrodes, respectively. To prepare the working electrode, 5 mg of catalyst was dispersed in a mixture of 960 μl isopropanol and 40 μl Nafion solution (5 wt%, Sigma-Aldrich), and sonicated for at least 30 minutes to obtain a homogeneous catalyst ink. Subsequently, 200 μl of the ink was coated onto a 1.0 cm x 1.0 cm carbon fiber paper, resulting in a loading of approximately 0.2 mg cm^{-2}. All potentials were converted to reversible hydrogen electrode (RHE) potentials using the Nernst equation:

$$E(RHE) = E(Ag/AgCl) + 0.198 \quad V + 0.059 \times pH$$

Before each measurement, the electrolyte was deoxygenated with argon for 30 minutes. Polarization curves were recorded at a scan rate of 20 mV s^{-1}. Chronoamperometry (CA) tests were performed at different potentials for 1 hour, with continuous argon flow (20 sccm). Catalyst stability was evaluated through 30 consecutive electrolysis cycles at -0.7 V vs. RHE and 8 hours long-term potentiostatic test at 100 mA cm^{-2}.

For the flow cell scale-up investigations, continuous electrolysis was performed in a customized zero-gap membrane electrode assembly (MEA) reactor. The geometric active area of the MEA was fixed at 1.0 cm^2. The carbon paper loaded with catalysts serves as the cathode, while nickel foam acts as the anode, positioned on opposite sides of the Nafion 117 ion exchange membrane. The catholyte and anolyte were continuously circulated through their respective flow field plates using a dual-channel peristaltic pump (or syringe pump). All MEA electrochemical tests, including polarization curves and long-term stability electrolysis, were conducted without internal iR compensation unless otherwise stated.

All electrochemical performances, including Faradaic efficiencies (FEs) and product yield rates, were evaluated based on at least three independent experiments under identical conditions. The data are presented as the mean values, and the error bars represent the standard deviation (SD).

6. Product analysis:

The liquid products were identified and quantified using ^1H NMR spectroscopy (Bruker Neo Nanobay 400 MHz). After a specified period of electrolysis, the electrolyte was collected from the cathodic compartment. To prepare the NMR sample, 0.5 mL of the post-reaction electrolyte was mixed with 0.1 mL of deuterium oxide for field-frequency locking and 0.1 mL of an internal standard solution containing TMSPA-d_4-Na. The identification of cyclohexanone oxime was confirmed by comparing the chemical shifts of its characteristic protons with those of high-purity standard samples in the same electrolyte environment. Quantification was performed by integrating the specific resonance peaks of the product relative to the singlet peak of TMSPA-d_4-Na = 0 ppm. A series of standard solutions with known concentrations of cyclohexanone oxime were prepared to establish a calibration curve for accurate quantification (**Figure S11**).

7. Determination and quantitation of NH_4^+

Using the salicylic acid-hypochlorous acid indophenol reaction, with sodium nitroprusside as a catalyst, the concentration of NH_4^+ in electrolytes was quantitatively determined by UV-visible detection at 655 nm. Specifically, solution A contained 0.5 M NaOH, 5 wt% sodium citrate, and 5 wt% salicylic acid. Solution B was a 0.05 M NaClO solution. Solution C was a 1 wt% sodium nitroprusside solution. After diluting the post-reaction electrolyte to an appropriate concentration, mix 2 mL of electrolyte, 2 mL of Solution A, 1 mL of Solution B, and 0.2 mL of Solution C. After standing at room temperature in the dark for 60 minutes, the absorbance of the solution was measured. Prepare solutions at different NH_4^+ concentrations (0.5 mg-N L^{-1}, 1 mg-N L^{-1}, 2 mg-N L^{-1}, 4 mg-N L^{-1}, 6 mg-N L^{-1}, 8 mg-N L^{-1}) and plot a calibration curve. The NH_4^+ concentration in the electrolyte was then determined using this calibration curve (**Figure S12**).

8. Calculation of product yield rate, Faradaic efficiency and selectivity:

The yield rate was calculated according to the following equation:

$$Yield\ rate = \frac{c \times V}{t \times S}$$

The FE was calculated according to the following equation:

$$FE(\%) = \frac{n \times F \times c \times V}{Q} \times 100\%$$

The selectivity was calculated according to the following equation:

$$Selectivity = \frac{Moles\ of\ formed\ cyclohexanone\ oxime}{Moles\ of\ consumed\ cyclohexanone}$$

Here, *n* is the number of electrons transferred for the formation of one cyclohexanone oxime molecule (e.g., n = 4 for the reduction of nitrite to the hydroxylamine-mediated product), *c* is the concentration of the product, *F* is the Faraday constant (96485 C mol^{-1}), *V* is the total volume of the electrolyte, *S* is the electrode area (1 cm^2), t is the electrolysis time, and *Q* is the total charge passed during electrolysis.

9. Electrochemical *in situ* attenuated total reflection Fourier transform infrared (ATR-FTIR) spectroscopy experiments:

Electrochemical *in situ* attenuated total reflection Fourier transform infrared (ATR-FTIR) spectroscopy experiments were conducted in a single-compartment PEEK spectroelectrochemical cell integrated with a VeeMAXIII ATR accessory. A standard three-electrode system was employed: a graphite rod as the counter electrode, a saturated Ag/AgCl electrode as the reference electrode, and the electrochemical process was precisely controlled by an Autolab potentiostat (PGSTAT204, Metrohm). Infrared spectra were collected using a Thermo Nicolet iS50 FTIR spectrometer equipped with a liquid nitrogen-cooled MCT detector. To achieve surface enhancement and improve signal quality, a gold (Au) thin film working electrode was prepared on the surface of a silicon (Si) ATR crystal prism with an incident angle of 60° using a modified chemical deposition method. Experiments were performed in 0.5 M KOH + 0.5 M KNO_2 and in a mixed electrolyte containing 0.1 M CYC; at specific potentials,

each spectrum was an average of 64 scans to ensure a sufficient signal-to-noise ratio. All spectra were acquired in single-beam mode. Background spectra were collected at the open-circuit potential (OCP) prior to the application of the cathodic potential. The reported spectra were obtained by subtracting the OCP background spectra.

10. *In situ* Raman measurements:

In situ electrochemical Raman measurements were performed in a single-compartment PEEK spectroelectrochemical cell equipped with a quartz window, using an inverted Raman microscope system. A 785 nm excitation source was precisely focused onto the electrode/electrolyte interface through a long working distance objective (50×, NA≈0.5). To suppress potential photothermal effects on the sample, the laser power reaching the sample surface was strictly controlled to below 0.5 mW. Electrochemical measurements were conducted using a standard three-electrode system: a graphite rod as the counter electrode, a saturated Ag/AgCl electrode as the reference electrode, and precise potential control was achieved using an Autolab potentiostat. The experiments were performed in 0.5 M KOH+ 0.5 M KNO_2+ 0.1 M CYC electrolyte, with a spectral acquisition range of 200-1800 cm^{-1} and a nominal resolution of 2 cm^{-1}. All spectral data were frequency calibrated using the characteristic peak of single-crystal silicon at 520.5 cm^{-1} before acquisition.

11. Electrochemical active surface area evaluation:

The electrochemical active surface area (ECSA) was determined by measuring the double-layer charging capacitance current using cyclic voltammetry (CV) at different scan rates within the non-Faradaic potential range. In a typical measurement experiment, the cyclic voltammetry potential window for the catalysts was set to 0.05 to 0.15 V (vs. RHE), and the electrolyte solution used was 0.5 M KOH. The set scan rate gradients were 10, 20, 40, 60, 80, and 100 mV s^{-1}. The double-layer capacitance (Cdl) was determined by plotting the cathodic charging current measured at the intermediate potential (0.1 V vs. RHE) as a function of the scan rate

and performing a linear fit. The average slope of this linear fit curve represents the Cdl value. The ECSA of the catalyst can be calculated by dividing the measured Cdl by the specific capacitance of the sample. In this experiment, the average specific capacitance was taken as 0.040 mF cm^{-2}. In accordance with established conventions for metal electrodes in alkaline electrolytes, this study adopted a specific capacitance value of Cs = 0.040 mF cm^{-2}, a value consistent with those employed in recent ECSA analyses of Bi-based electrocatalysts[5-6].

Computational details

Density functional theory (DFT) calculations were performed using the Vienna Ab initio Simulation Package (VASP)[7-8], with workflows implemented through the Atomic Simulation Environment (ASE)[9]. The exchange-correlation functional was described by the RPBE functional[10], and core-valence interactions were treated using the projector augmented-wave (PAW) method. A plane-wave kinetic energy cutoff of 450 eV was used throughout. Electronic
s
e
l
f
-
c
o
n
s
i
s
t
e
n
c
y

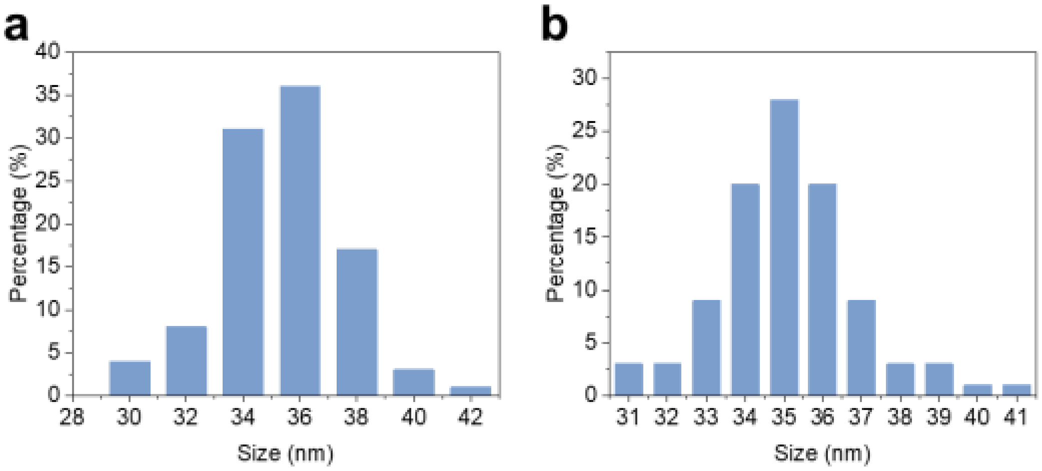

Figure S1. The particle size distribution of Bi RDs (a) and Bi NPs (b).

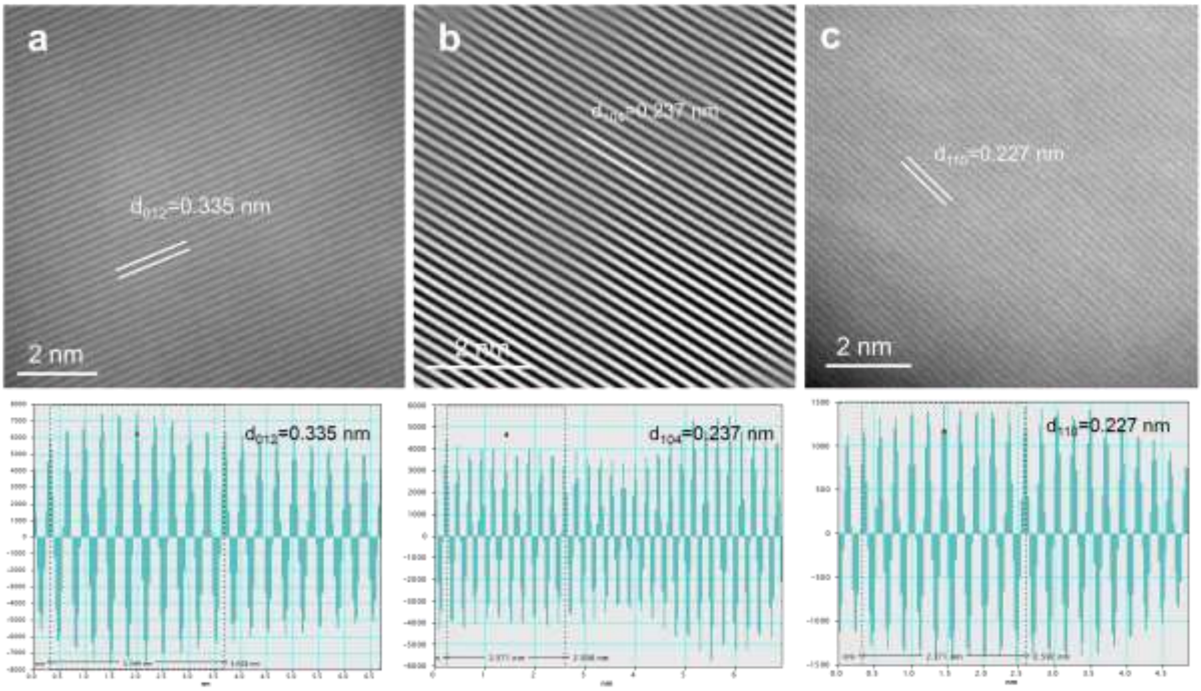

Figure S2. HRTEM images of Bi RDs. (a-c) High-magnification HRTEM images showing clear lattice fringes corresponding to the (012), (104), and (110) planes, respectively. The bottom panels display the corresponding intensity line profiles measured across multiple lattice fringes, confirming average interplanar spacings of 0.335, 0.237, and 0.227 nm.

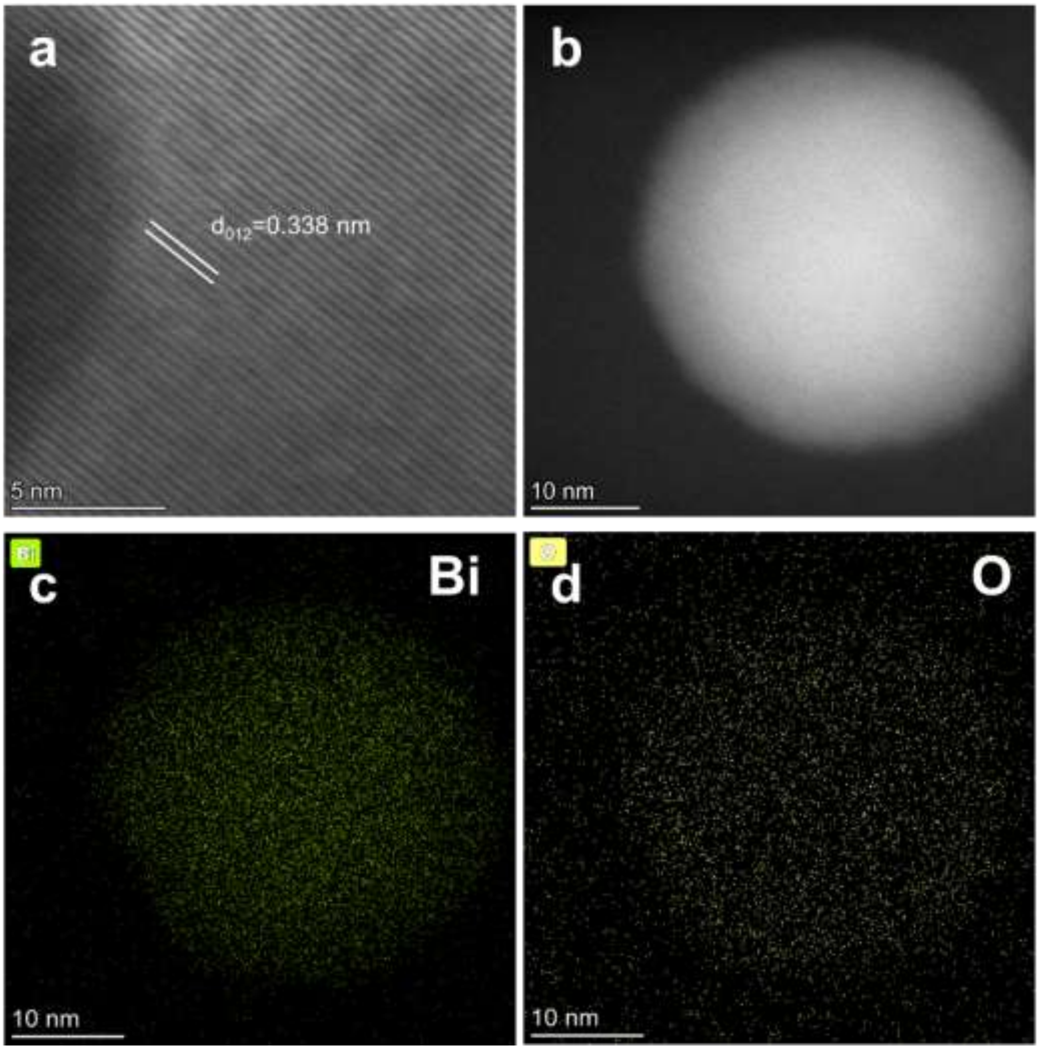

Figure S3. Characterization of as-synthesized Bi NPs. (a) HRTEM image of Bi NPs, showing clear lattice fringes with an interplanar spacing of 0.338 nm, corresponding to the (012) plane. (b-d) HAADF-STEM image and the corresponding EDS elemental mapping images of Bi and O.

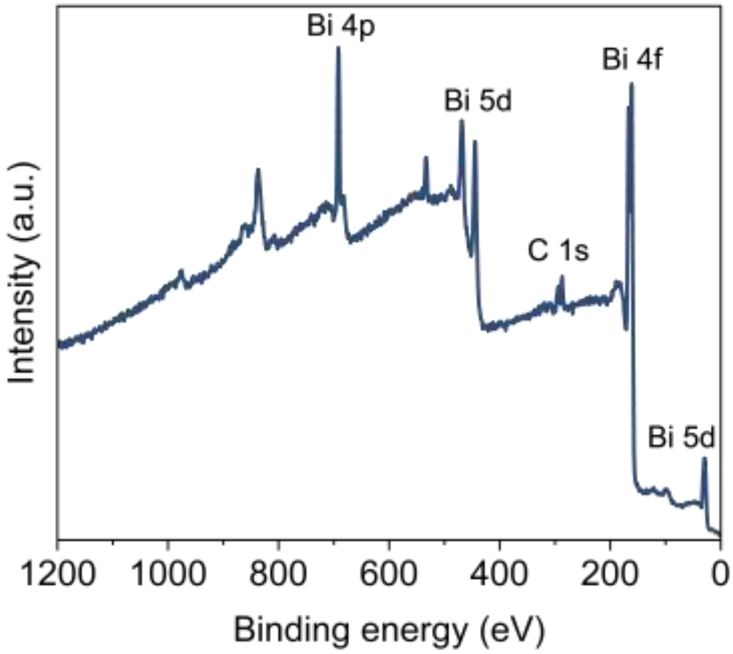

Figure S4. XPS survey spectrum of the Bi RDs. The spectrum displays the characteristic peaks of Bi 4f, Bi 4p, and Bi 5d, confirming the chemical composition of the synthesized Bi RDs. The C 1s peak at ~284.8 eV is attributed to adventitious carbon contamination.

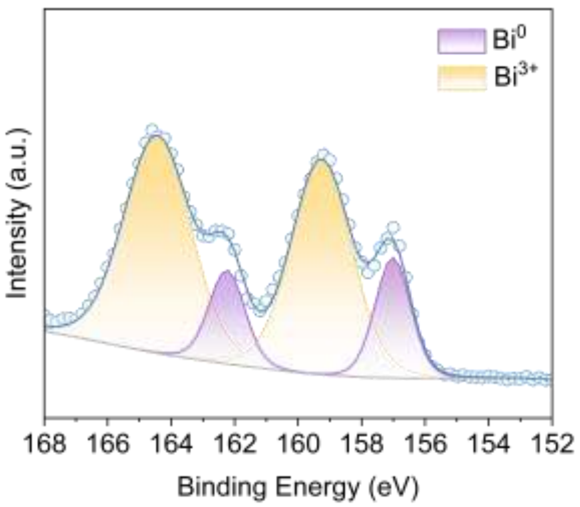

Figure S5. High-resolution spectrum of Bi 4f electrons of Bi NPs.

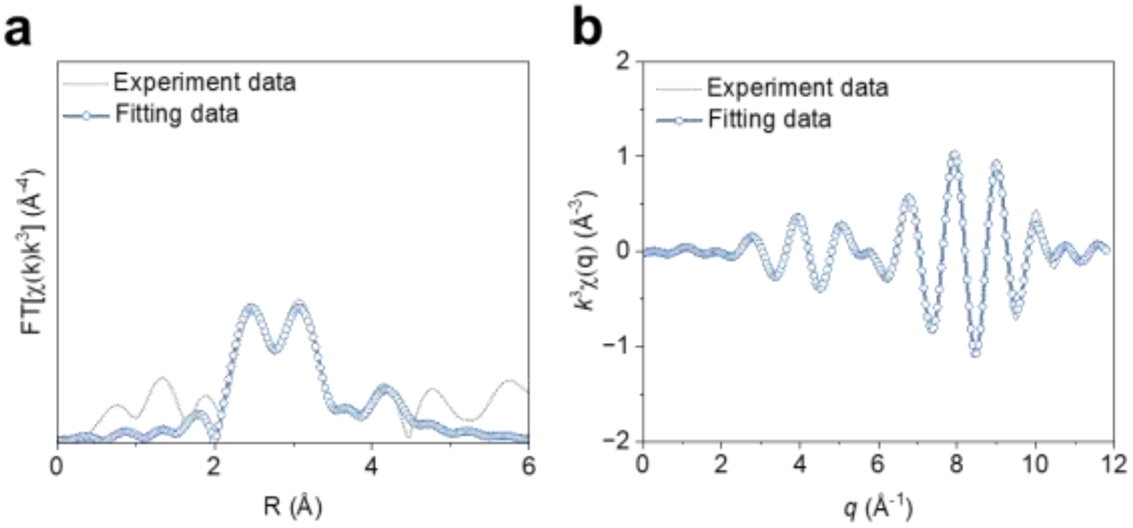

Figure S6. EXAFS fitting results for the Bi RDs. (a) The magnitude of the Fourier transformed (FT) k^3-weighted EXAFS signal in R-space. (b) The corresponding Fourier filtered k^3-weighted EXAFS oscillations in q-space. The experimental data and best-fit models are displayed as solid gray lines and open blue circles, respectively.

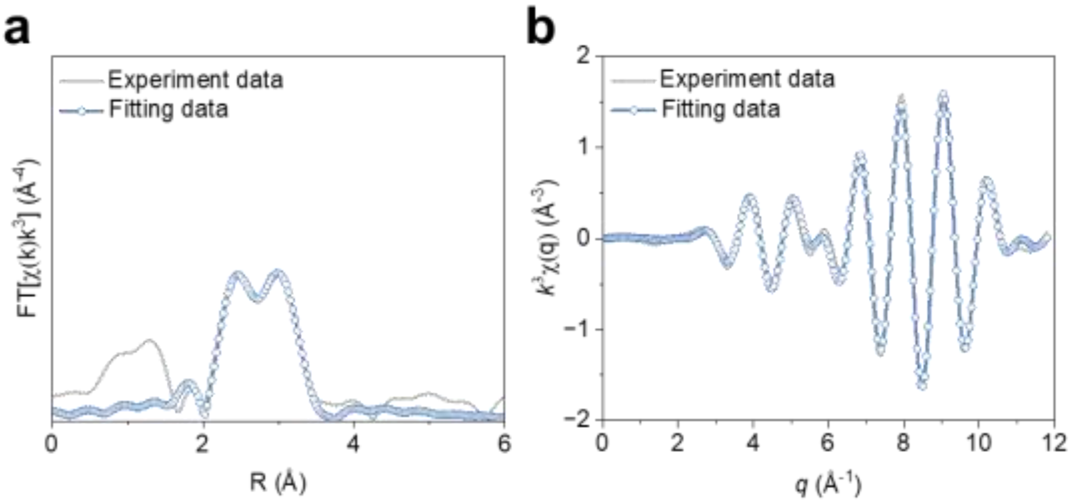

Figure S7. EXAFS fitting results for the Bi nanoparticles. (a) The magnitude of the Fourier transformed (FT) k^3-weighted EXAFS signal in R-space. (b) The corresponding Fourier filtered k^3-weighted EXAFS oscillations in q-space. The experimental data and best-fit models are displayed as solid gray lines and open blue circles, respectively.

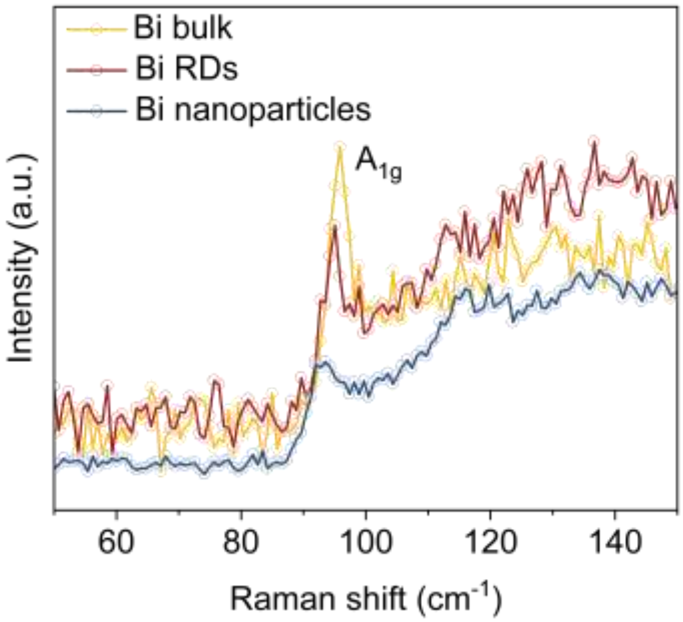

Figure S8. Raman spectra of commercial Bi bulk, Bi RDs and Bi nanoparticles.

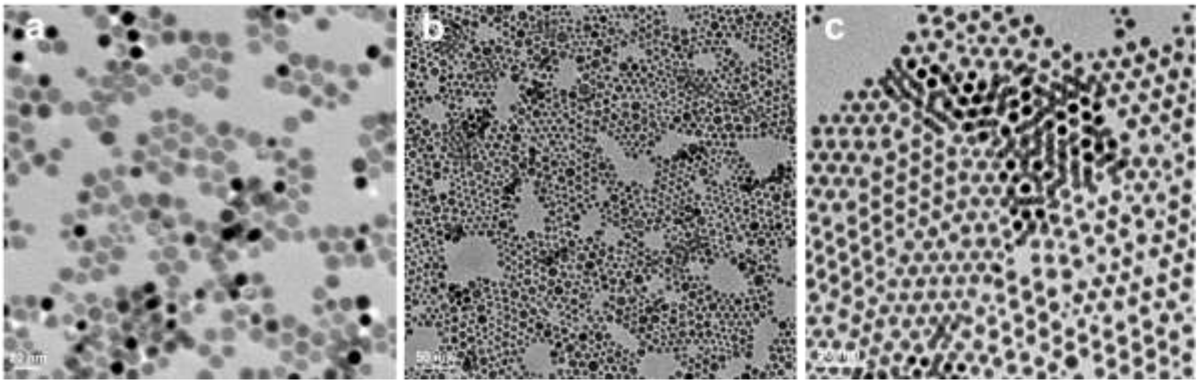

Figure S9. (a)TEM image of Cu NPs (~11 nm). (b)TEM image of Ag NPs (~12 nm). (c)TEM image of Pd NPs (12 nm).

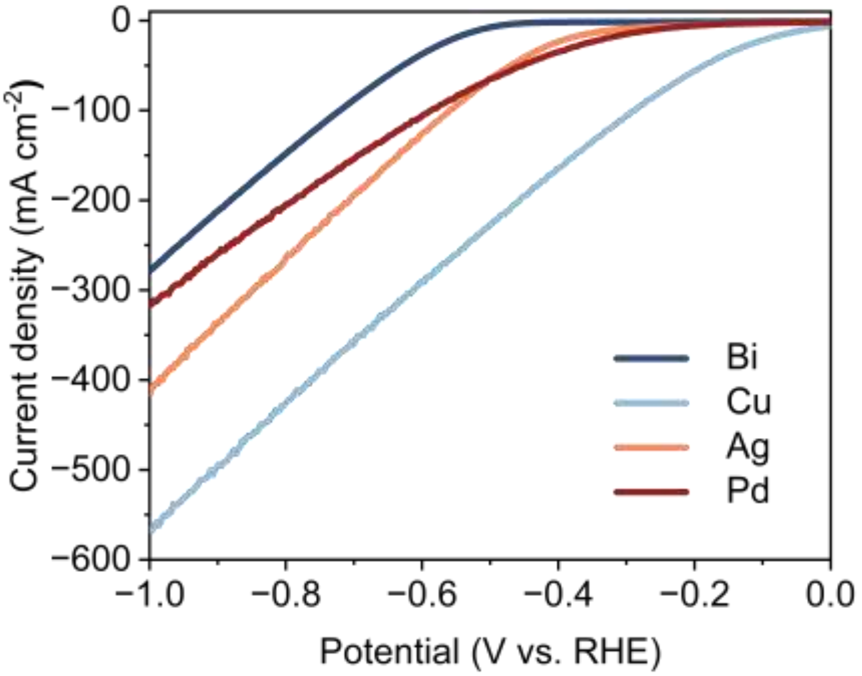

Figure S10. Electrocatalytic performance comparison. Linear sweep voltammetry (LSV) curves of the Bi RDs, Cu nanoparticles, Ag nanoparticles, and Pd nanoparticles electrodes recorded in 0.5 M KOH+0.5 M KNO_2+0.1 M CYC at a scan rate of 20 mV s^{-1}. The Cu catalyst exhibits the highest current density across the potential range, while Bi shows the lowest cathodic current response.

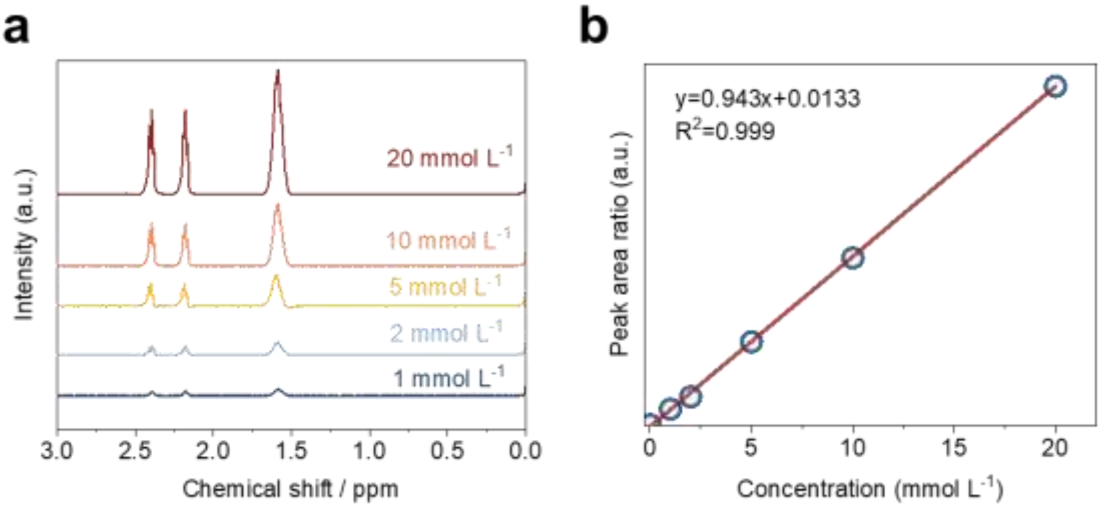

Figure S11. Quantitative analysis of cyclohexanone oxime by ^{1}H NMR using an internal standard. (a) ^{1}H NMR spectra of CHO standard solutions with concentrations ranging from 1 to 20 mmol L^{-1}. (b) The corresponding standard calibration curve plotting the integrated peak area ratio against the concentration. The high correlation coefficient ($R^2 = 0.999$) of the linear regression ($y = 0.943x + 0.0133$) confirms the reliability of the quantification method.

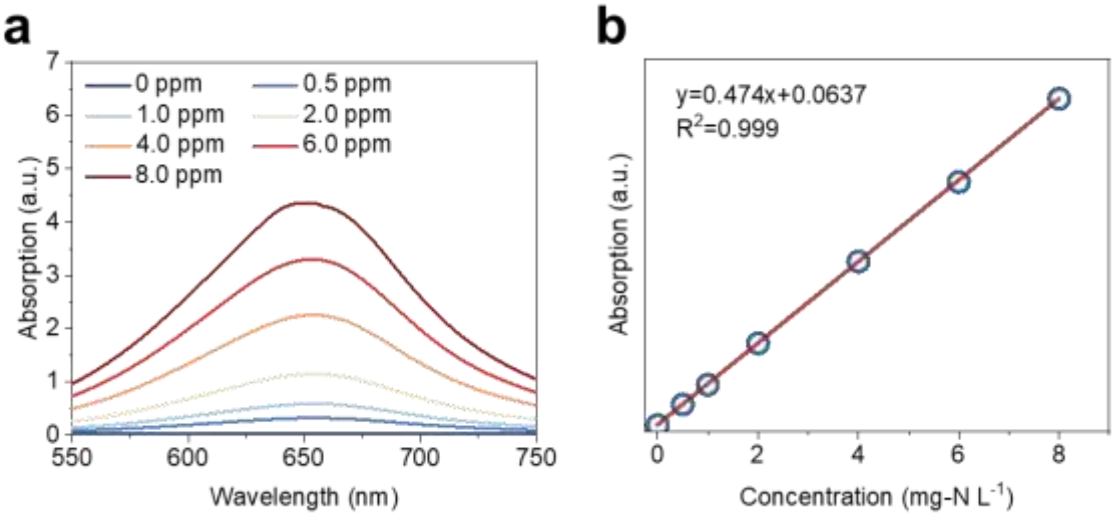

Figure S12. UV-vis measurements of the concentrations of ammonium with a modified indophenol blue method. (a) The UV-visible absorption spectra of different solutions with different NH_4^+ concentrations. (b) Calibration curve colorimetric NH_4^+ assay using modified indophenol blue method. Electrolytes after electrolysis were diluted to ensure the NH_4^+ concentrations in the test solutions were in the linear range of the calibration curve.

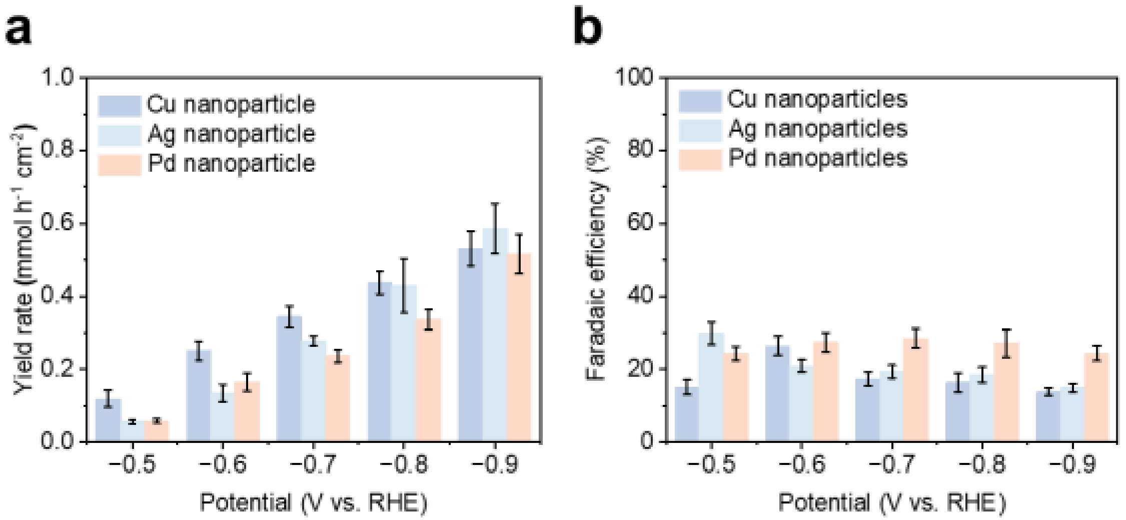

Figure S13. Performance evaluation of electrocatalytic CHO synthesis. (a) CHO yield rate on Cu NPs, Ag NPs and Pd NPs at different applied potentials in 0.5 M KOH+0.5 M KNO_2+0.1 M CYC. (b) FE of CHO on Cu NPs, Ag NPs and Pd NPs at different applied potentials (vs. RHE) in 0.5 M KOH+0.5 M KNO_2+0.1 M CYC.

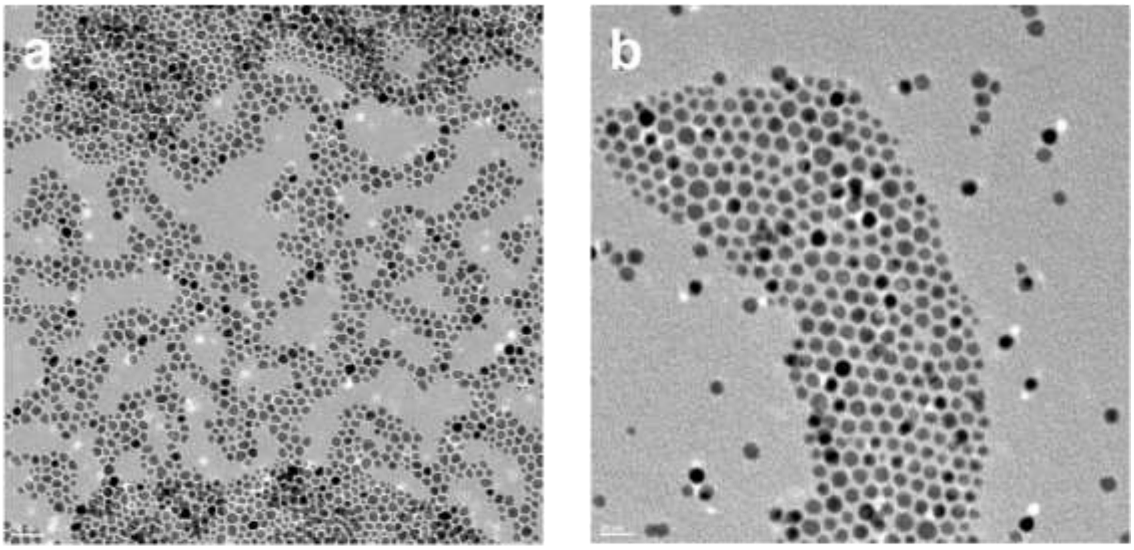

Figure S14. (a)TEM image of SnO_x nanoparticles. (b)TEM image of In_2O_3 nanoparticles.

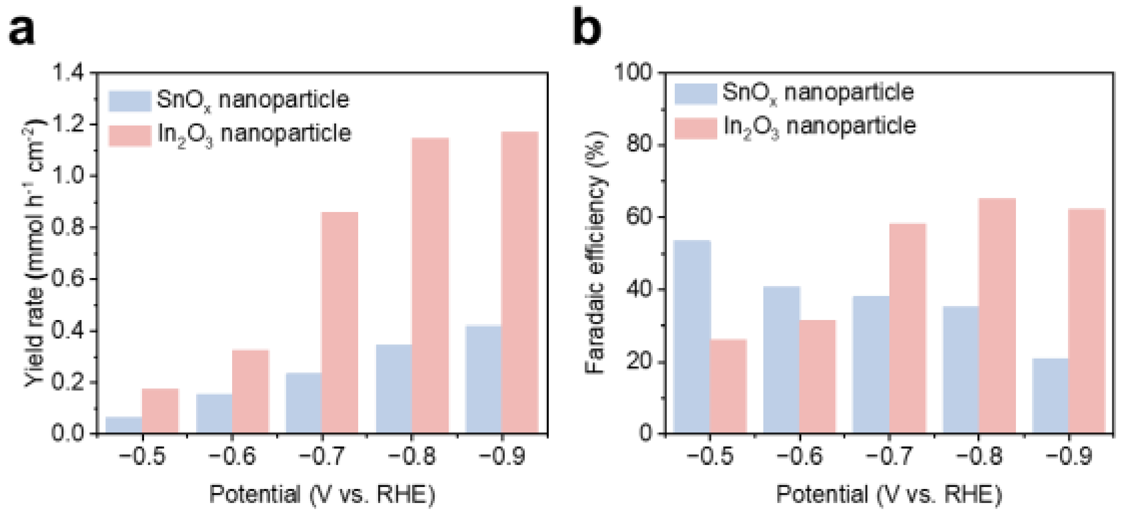

Figure S15. Performance evaluation of electrocatalytic CHO synthesis. (a) CHO yield rate on SnO_x and In_2O_3 at different applied potentials in 0.5 M KOH+0.5 M KNO_2+0.1 M CYC. (b) FE of CHO on SnO_x and In_2O_3 at different applied potentials (vs. RHE) in 0.5 M KOH+0.5 M KNO_2+0.1 M CYC.

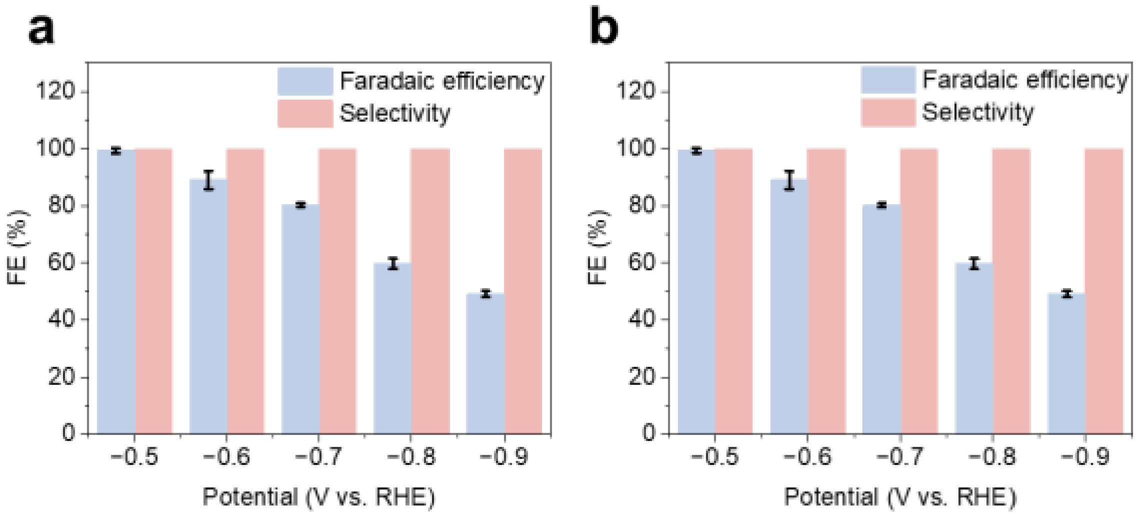

Figure S16. Performance evaluation of electrocatalytic cyclohexanone oxime synthesis. (a) FE and selectivity of CHO on Bi RDs at different applied potentials. (b) FE and selectivity of CHO on Bi NPs at different applied potentials.

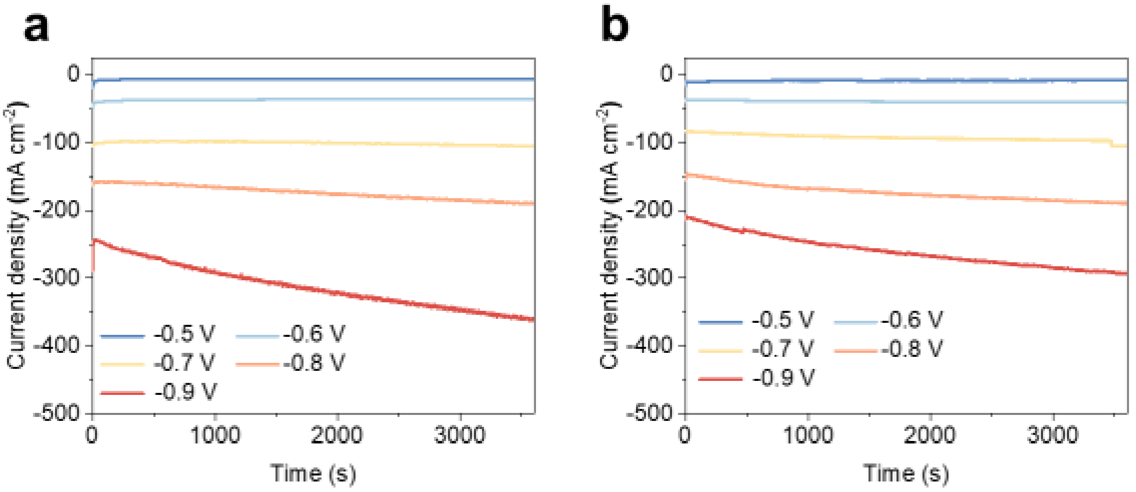

Figure S17. Electrochemical synthesis of CHO. (a) Chronoamperometric (i-t) curves of the Bi RDs electrode recorded at different applied potentials ranging from -0.5 to -0.9 V vs. RHE. (b) Chronoamperometric curves of the Bi NPs electrode recorded under the same potential range.

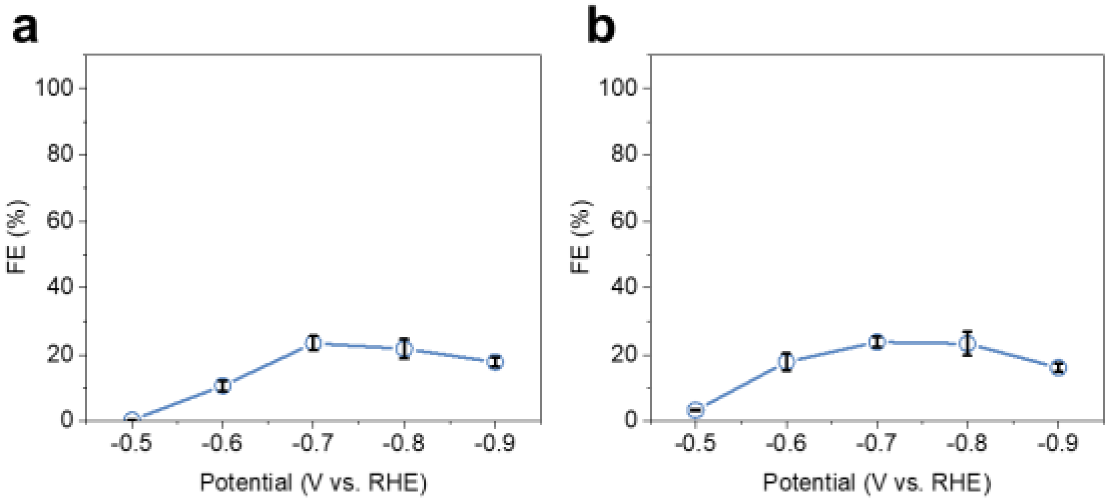

Figure S18. Evaluation of the side reaction. (a) FE of the NH_3 byproduct generated on the Bi RDs electrode at applied potentials ranging from -0.5 to -0.9 V vs RHE. (b) FE of the NH_3 byproduct generated on the Bi NPs electrode at applied potentials ranging from -0.5 to -0.9 V vs RHE.

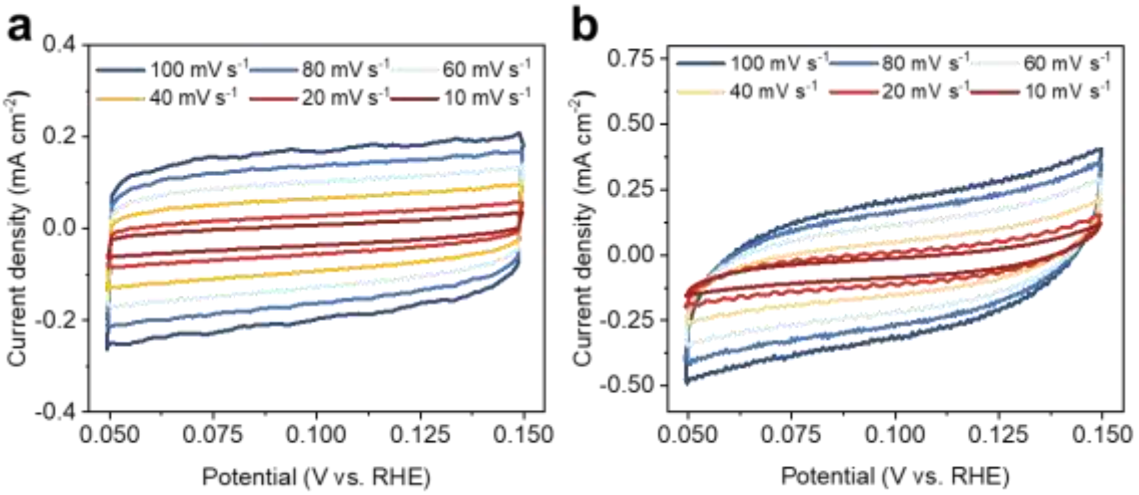

Figure S19. Cyclic voltammogram curves of (a) Bi RDs, (b) Bi NPs in Ar-saturated 0.5 M KOH from 0.05 to 0.15 V vs. RHE with scan rates of 10, 20, 40, 60, 80, and 100 mV s^{-1}.

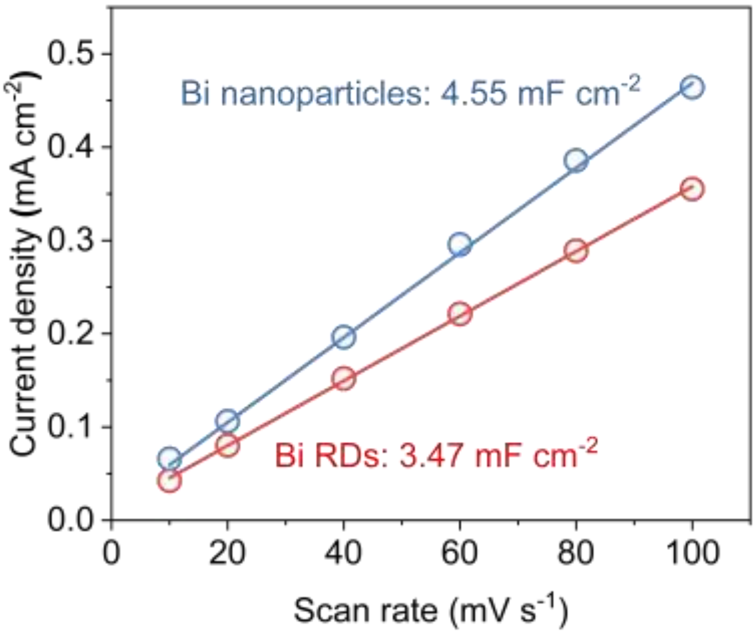

Figure S20. The linear relationship between the current densities at 0.1 V vs. RHE and scan rate.

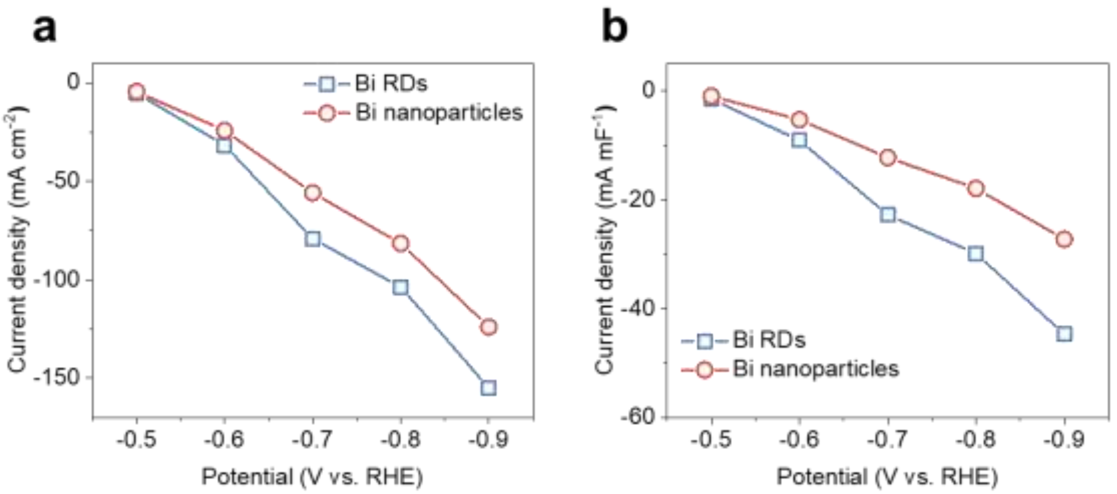

Figure S21. (a) The geometric area normalized partial current densities of CHO on Bi RDs and Bi NPs in the H-type cell. (b) The ECSA normalized partial current densities of CHO on Bi RDs and Bi NPs in the H-type cell.

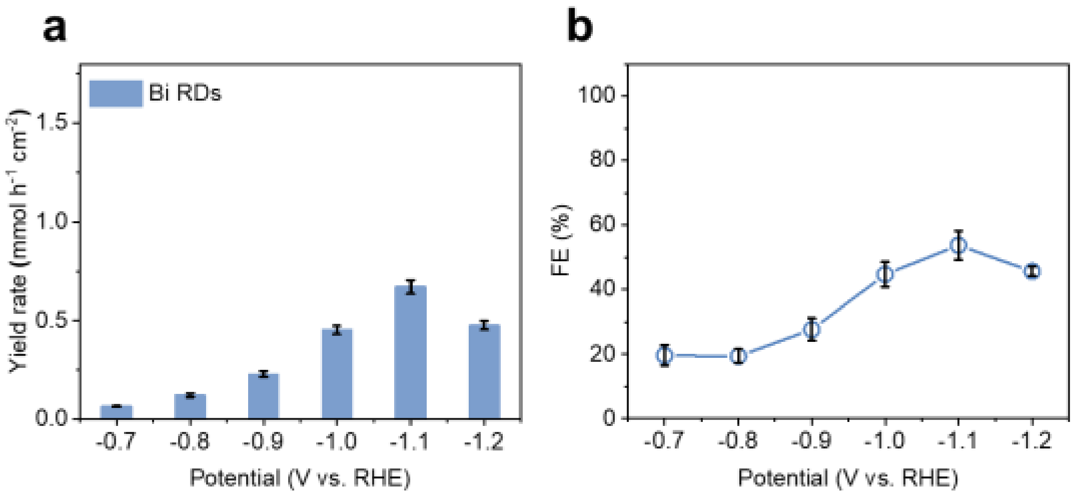

Figure S22. Performance evaluation of electrocatalytic CHO synthesis. (a) CHO yield rate on Bi RDs at different applied potentials in 0.5 M PBS +0.5 M KNO_2+0.1 M CYC. (b) FE of CHO on Bi RDs at different applied potentials in 0.5 M PBS+0.5 M KNO_2+0.1 M CYC.

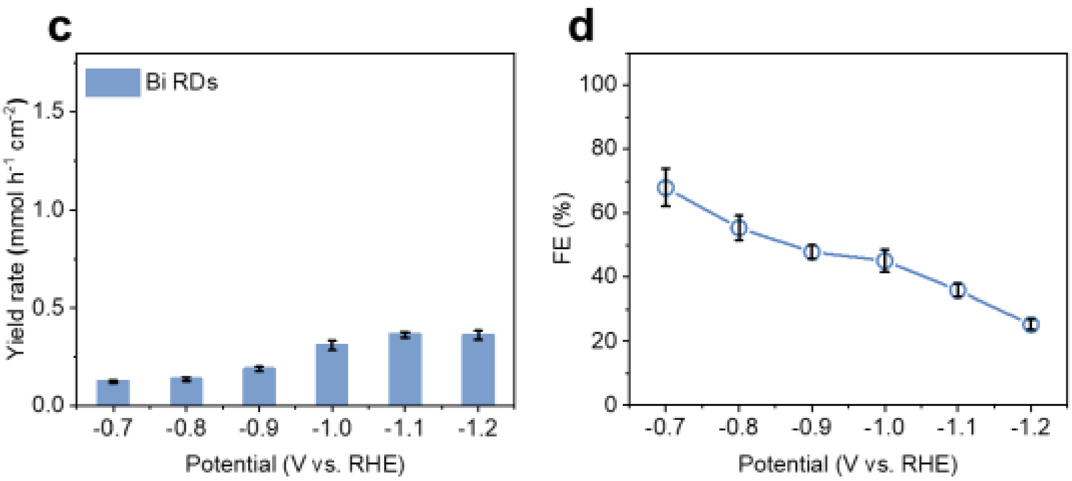

Figure S23. Performance evaluation of electrocatalytic CHO synthesis. (a) CHO yield rate on Bi RDs at different applied potentials in 0.5 M $KHCO_3$+0.5 M KNO_2+0.1 M CYC. (b) FE of CHO on Bi RDs at different applied potentials in 0.5 M $KHCO_3$+0.5 M KNO_2+0.1 M CYC.

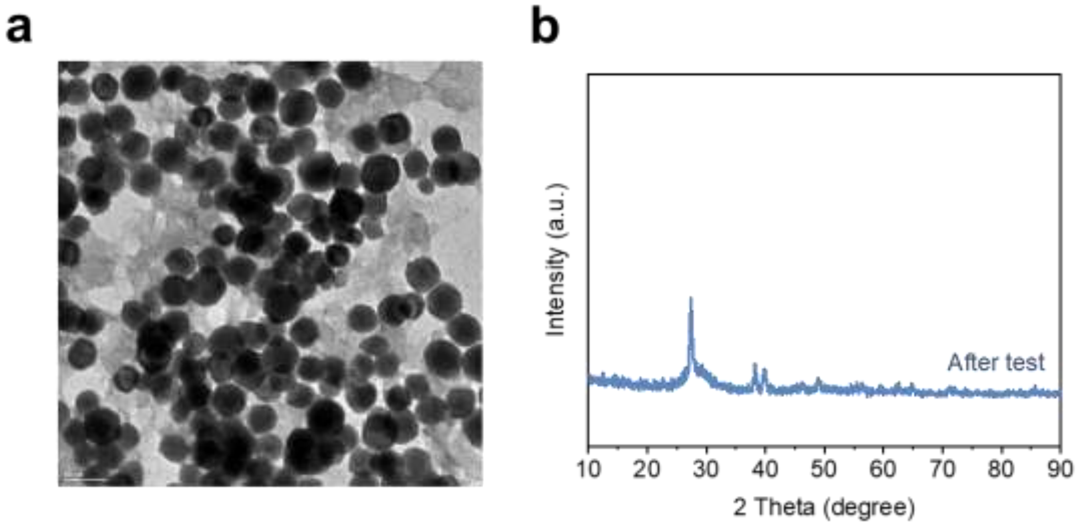

Figure S24. (a) TEM image of Bi RDs after electrochemical test. (b) XRD pattern of Bi RDs after electrochemical test.

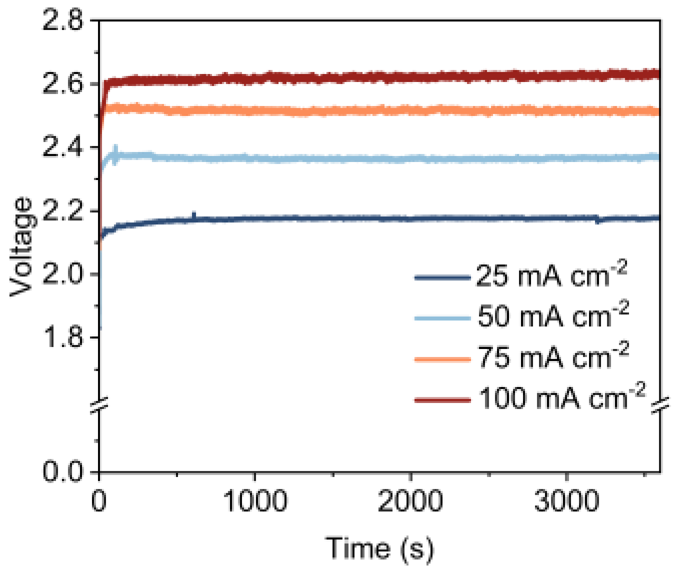

Figure S25. Chronopotentiometry recorded during the continuous electrosynthesis of CHO for 1 h at constant current densities of 25, 50, 75, and 100 mA cm^{-2} in flow cell.

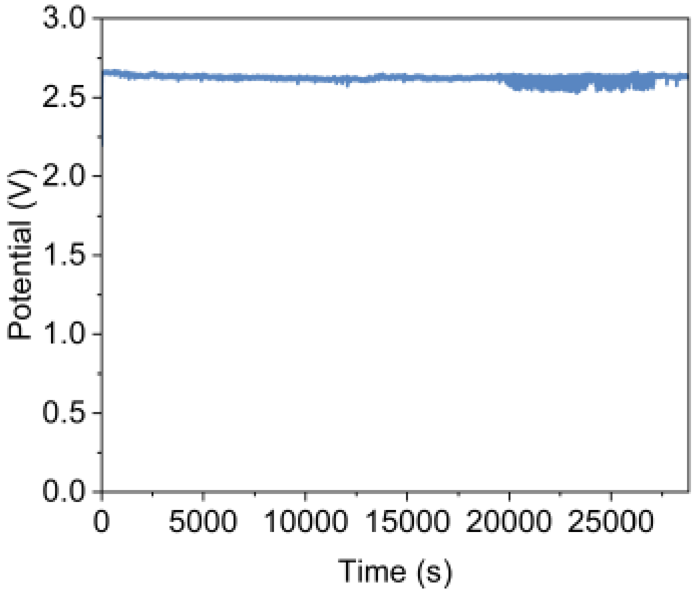

Figure S26. The cell voltage profile recorded during the continuous electrosynthesis of CHO for 8 h at a constant current density of 100 mA cm^{-2} in flow cell.

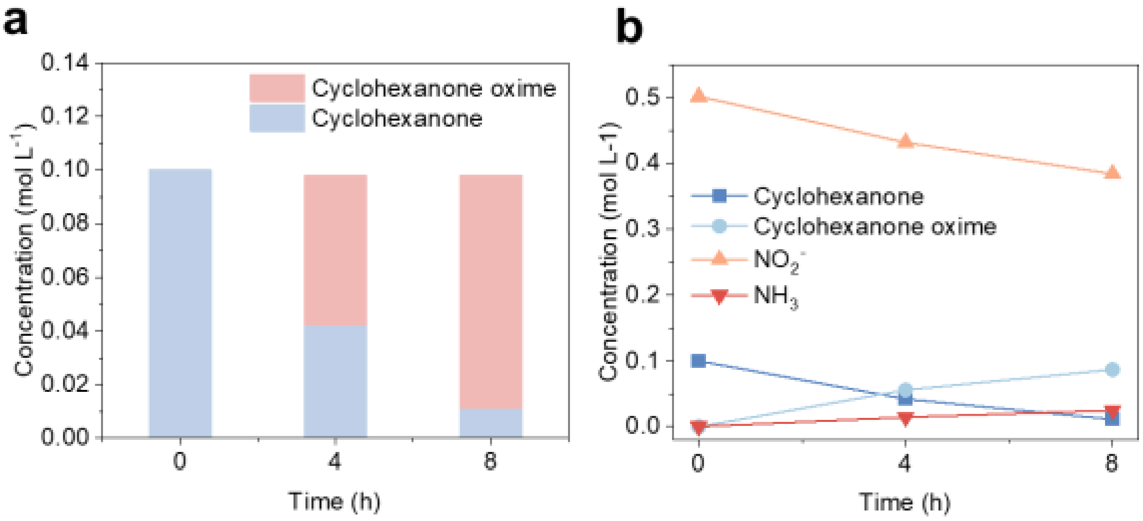

Figure S27. Carbon and nitrogen mass balance during 8 h flow cell electrolysis. (a) Concentrations of CYC and CHO at 0, 4, and 8 h (b) Time evolution of CYC, CHO, NO_2^-, and NH_3 concentrations.

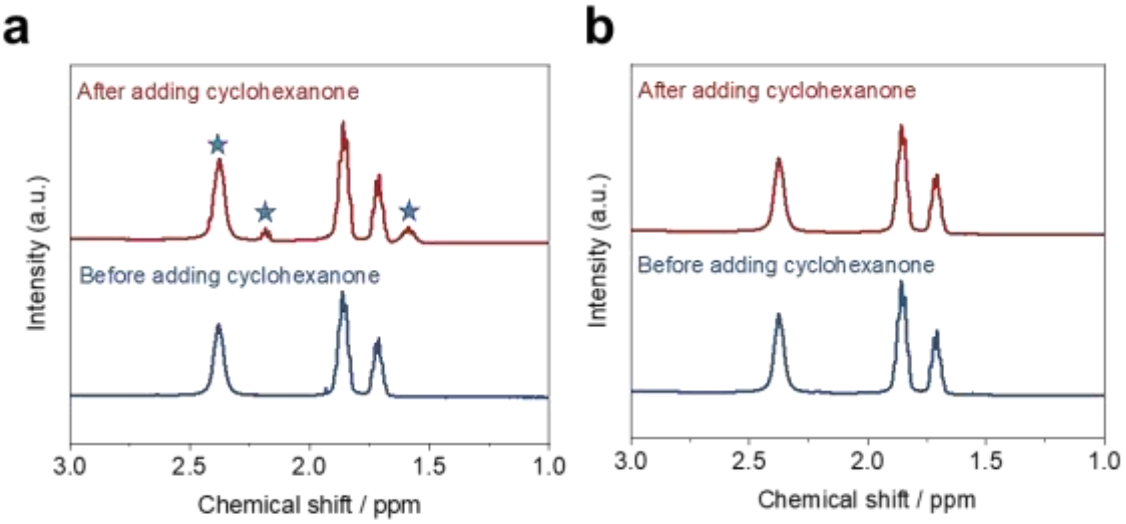

Figure S28. (a)^{1}H NMR of Bi RDs catalyzed electrolyte (0.5 M KOH+0.5 M KNO_2) before and after adding CYC. (b)^{1}H NMR of Cu nanoparticles catalyzed electrolyte (0.5 M KOH+0.5 M KNO_2) before and after adding CYC.

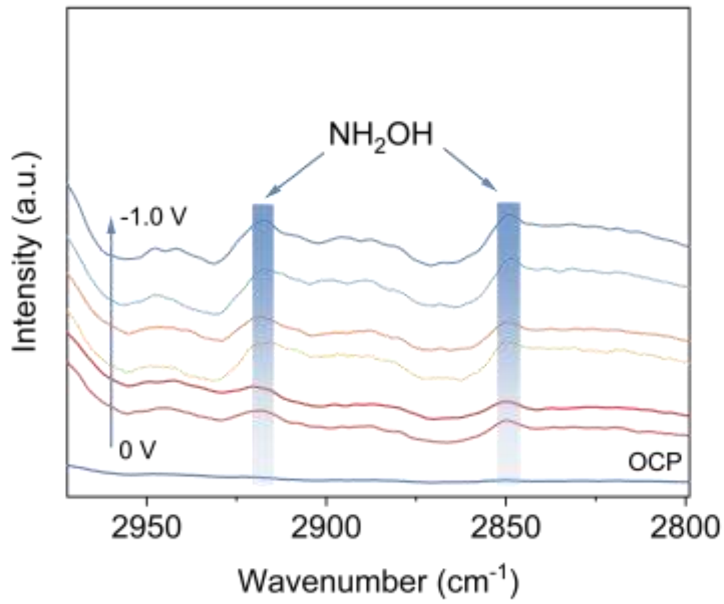

Figure S29. *In situ* ATR-FTIR spectra of Bi RDs in the electrolyte containing 0.5 M KOH and 0.5 M KNO_2.

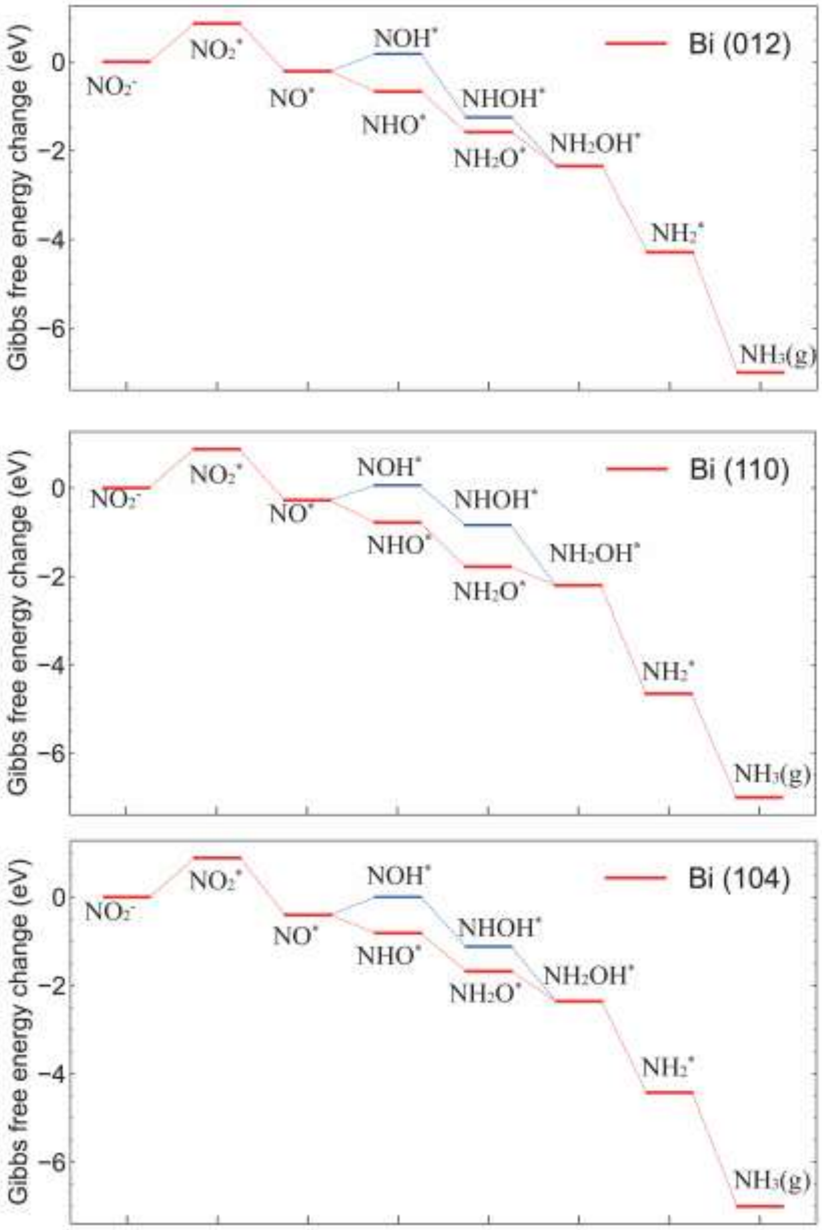

Figure S30. Reaction pathways of NO_2RR to NH_3 on Bi. (a) Free energy profile for electrochemical reduction of NO_2^- to NH_3 on Bi(012) at -0.5 V vs. RHE from GC-DFT calculations. (b) Free energy profile for electrochemical reduction of NO_2^- to NH_3 on Bi(110) at -0.5 V vs. RHE from GC-DFT calculations. (c) Free energy profile for electrochemical reduction of NO_2^- to NH_3 on Bi(104) at -0.5 V vs. RHE from GC-DFT calculations. Two different pathways are shown for all surfaces where the pathways bifurcate after the hydrogenation of *NO.

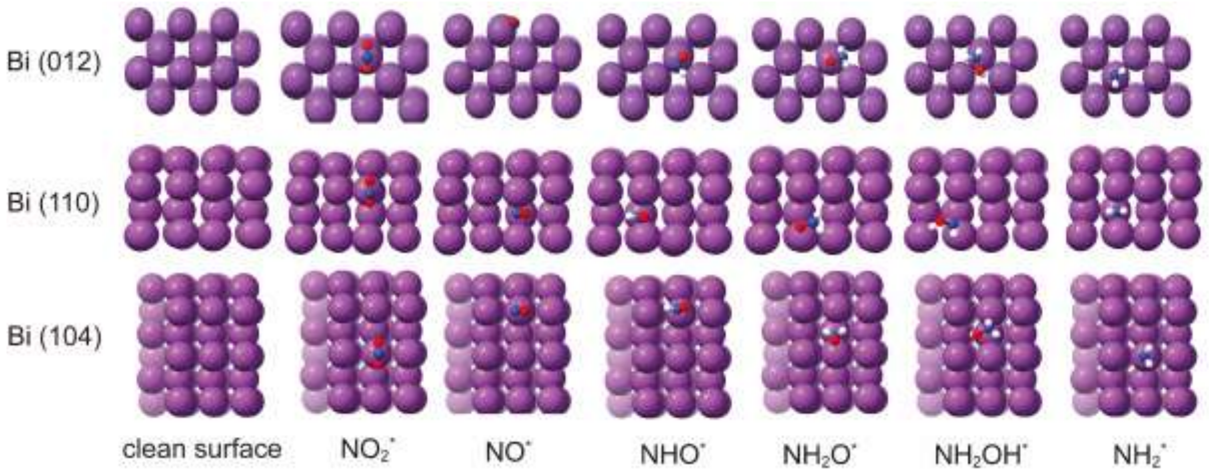

Figure S31. Adsorption configurations of reaction intermediates (top view) are shown (Bi: purple, N: blue, O: red, H: white).

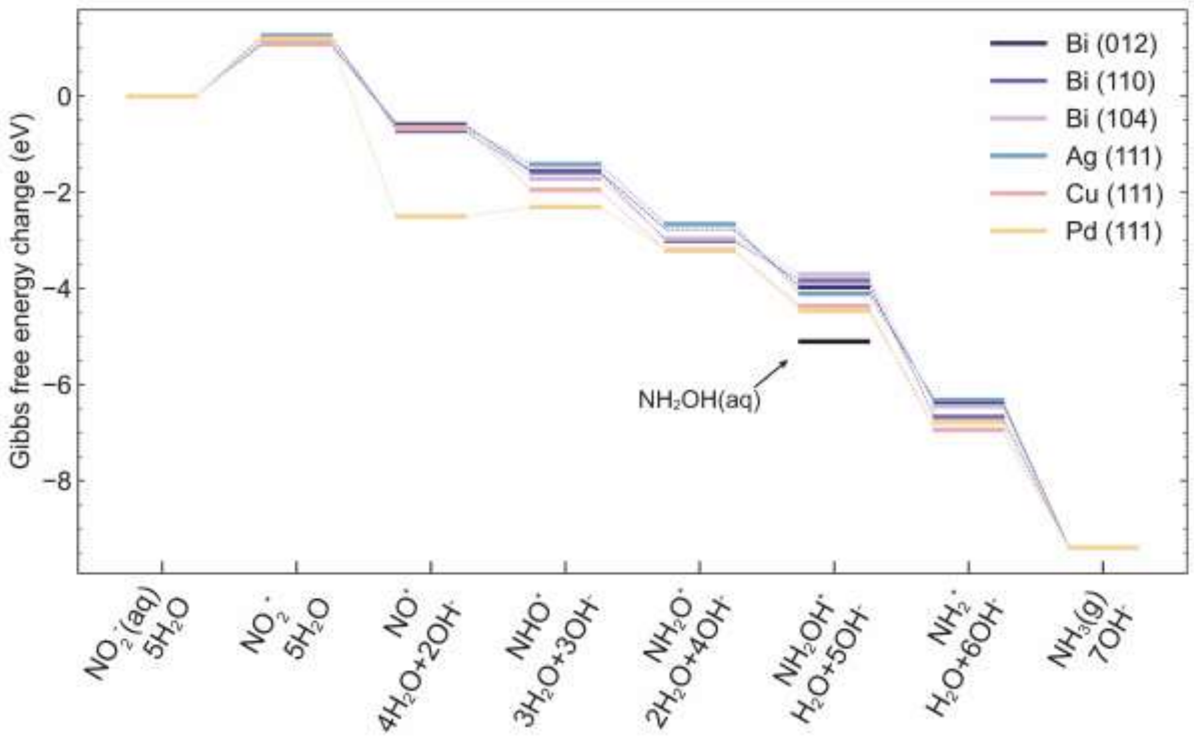

Figure S32. Free energy profiles of NH_3 formation from NO_2^- reduction at -0.9 V vs. RHE from grand canonical DFT calculations. The free formation energy of intermediate products NH_2OH(aq) is marked.

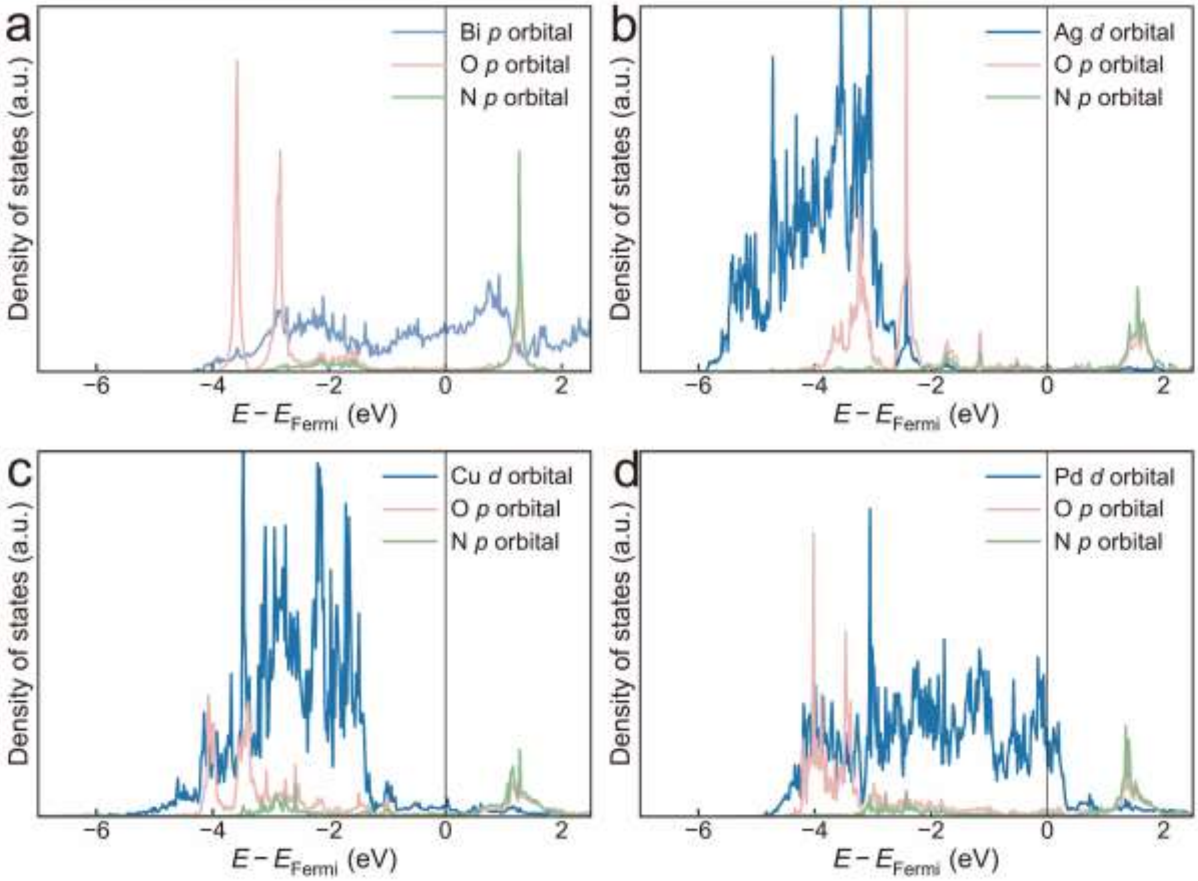

Figure S33. PDOS of adsorbed NO_2 on the (a) Bi, (b) Ag, (c) Cu and (d) Pd surfaces.

Table S1. EXAFS fitting parameters at the Bi L3-edge for various samples (S_0^2=0.85[a] from Bi-foil).

Sample	Scattering pair	CN[b]	R(Å)[c]	σ^2(Å^2)[d]	ΔE_0(eV)[e]	R factor
Bi RDs	Bi-Bi	2.7±0.4	3.06±0.1	0.0053 ±0.0005	-3.1±1.2	0.0075
Bi NPs	Bi-Bi	2.5±0.4	3.06±0.1	0.0072 ±0.0008	-4.3±1.4	0.0088
Bi foil	Bi-Bi	3	3.07±0.01	0.0051	-3.3±0.8	0.0058

[a] S_0^2: is the amplitude reduction factor, determined by the fitting of Bi foil;

[b] *CN*: coordination numbers;

[c] *R*: bond distance;

[d] σ^2: Debye-Waller factors;

[e] ΔE_0: the inner potential correction.

Error bounds that characterize the structural parameters obtained by EXAFS spectroscopy were estimated as CN±20%; R ± 1%; σ^2 ± 20%.

Table S2. Summary of the performance of electrocatalysts for the synthesis of CHO.

Electrocatalysts	Potentials (V vs. RHE)	Yield rate (mmol h^{-1} cm^{-2})	FE	Reactor	Reference
CuS	-0.9	0.165	26.1%	H cell	[12]
$Zn_{93}Cu_7$	-0.8	0.25	27%	H cell	[13]
PdCuAgBiIn	-0.9	0.288	47.6 %	H cell	[14]
Ag	100 mA cm^{-2}	0.78	83.8%	Flow cell	[15]
$Bi_{90}Mo_{10}$	-1.5	0.38	76%	H cell	[16]
$Cu_xC_yO_z$@600	-1.6	0.308	47.8%	H cell	[17]
Cu−Mo	200 mA cm^{-2}	3.0	85.2%	Flow cell	[18]
FeBPAbipyH	-0.4	0.769	77.3%	Flow cell	[19]
	-0.5	0.068	99.2%	H cell	
Bi RDs	-0.7	0.79	80.3%	H cell	This work
	-0.9	1.4	49.1%	H cell	

Table S3. Control experiments for CHO production.

Cathode	C source	N source	Electrolysis	Cyclohexanone oxime
Bi	cyclohexanone	KNO_2	Yes	Yes
Bi	cyclohexanone	KNO_2	No	Not Detected
Bi	cyclohexanone	NH_2OH	Yes	Yes
Bi	cyclohexanone	NH_2OH	No	Yes
Bi	cyclohexanone	NH_3	Yes	Not Detected
Bi	cyclohexanone	KNO_3	Yes	Yes
Bi	cyclohexanone	\	Yes	Not Detected
Bi	\	KNO_2	Yes	Not Detected
Carbon paper	cyclohexanone	KNO_2	Yes	Not Detected

Reference

1. Wei, B.; Zhang, X.; Zhang, C.; Jiang, Y.; Fu, Y. Y.; Yu, C.; Sun, S. K.; Yan, X. P., Facile Synthesis of Uniform-Sized Bismuth Nanoparticles for CT Visualization of Gastrointestinal Tract in Vivo. *ACS Appl. Mater. Interfaces* **2016,** *8* (20), 12720-6.
2. Yang, Y.; Louisia, S.; Yu, S.; Jin, J.; Roh, I.; Chen, C.; Fonseca Guzman, M. V.; Feijoo, J.; Chen, P. C.; Wang, H.; Pollock, C. J.; Huang, X.; Shao, Y. T.; Wang, C.; Muller, D. A.; Abruna, H. D.; Yang, P., Operando studies reveal active Cu nanograins for CO_2 electroreduction. *Nature* **2023,** *614* (7947), 262-269.
3. Liu, Q.; Bauer, J. C.; Schaak, R. E.; Lunsford, J. H., Supported palladium nanoparticles: an efficient catalyst for the direct formation of H_2O_2 from H_2 and O_2. *Angew. Chem. Int. Ed.* **2008,** *47* (33), 6221-4.
4. Ko, Y. J.; Lim, C.; Jin, J.; Kim, M. G.; Lee, J. Y.; Seong, T. Y.; Lee, K. Y.; Min, B. K.; Choi, J. Y.; Noh, T.; Hwang, G. W.; Lee, W. H.; Oh, H. S., Extrinsic hydrophobicity-controlled silver nanoparticles as efficient and stable catalysts for CO_2 electrolysis. *Nat. Commun.* **2024,** *15* (1), 3356.
5. McCrory, C. C.; Jung, S.; Peters, J. C.; Jaramillo, T. F., Benchmarking heterogeneous electrocatalysts for the oxygen evolution reaction. *J. Am. Chem. Soc.* **2013,** *135* (45), 16977-87.
6. Xie, H.; Zhang, T.; Xie, R.; Hou, Z.; Ji, X.; Pang, Y.; Chen, S.; Titirici, M. M.; Weng, H.; Chai, G., Facet Engineering to Regulate Surface States of Topological Crystalline Insulator Bismuth Rhombic Dodecahedrons for Highly Energy Efficient Electrochemical CO_2 Reduction. *Adv. Mater.* **2021,** *33* (31), e2008373.
7. Kresse, G.; Furthmüller, J., Efficient iterative schemes for ab initio total-energy calculations using a plane-wave basis set. *Phys. Rev. B: Condens. Matter* **1996,** *54* (16), 11169.
8. Kresse, G.; Furthmüller, J., Efficiency of ab-initio total energy calculations for metals and semiconductors using a plane-wave basis set. *Comput. Mater. Sci* **1996,** *6* (1), 15-50.
9. Hjorth Larsen, A.; Jorgen Mortensen, J.; Blomqvist, J.; Castelli, I. E.; Christensen, R.; Dulak, M.; Friis, J.; Groves, M. N.; Hammer, B.; Hargus, C.; Hermes, E. D.; Jennings, P. C.; Bjerre Jensen, P.; Kermode, J.; Kitchin, J. R.; Leonhard Kolsbjerg, E.; Kubal, J.; Kaasbjerg, K.; Lysgaard, S.; Bergmann Maronsson, J.; Maxson, T.; Olsen, T.; Pastewka, L.; Peterson, A.; Rostgaard, C.; Schiotz, J.; Schutt, O.; Strange, M.; Thygesen, K. S.; Vegge, T.; Vilhelmsen, L.; Walter, M.; Zeng, Z.; Jacobsen, K. W., The atomic simulation environment-a Python library for working with atoms. *J Phys Condens Matter* **2017,** *29* (27), 273002.
10. Hammer, B. H. L. B.; Hansen, L. B.; Nørskov, J. K., Improved adsorption energetics within density-functional theory using revised Perdew-Burke-Ernzerhof functionals. *Phys. Rev. B: Condens. Matter* **1999,** *59* (11), 7413.
11. Islam, S. M. R.; Khezeli, F.; Ringe, S.; Plaisance, C., An implicit electrolyte model for plane wave density functional theory exhibiting nonlinear response and a nonlocal cavity definition. *J. Chem. Phys.* **2023,** *159* (23).
12. Wu, Y.; Zhao, J.; Wang, C.; Li, T.; Zhao, B. H.; Song, Z.; Liu, C.; Zhang, B., Electrosynthesis of a nylon-6 precursor from cyclohexanone and nitrite under ambient conditions. *Nat. Commun.* **2023,** *14* (1), 3057.
13. Sharp, J.; Ciotti, A.; Andrews, H.; Udayasurian, S. R.; Garcia-Melchor, M.; Li, T., Sustainable Electrosynthesis of Cyclohexanone Oxime through Nitrate Reduction on a Zn-Cu Alloy Catalyst. *ACS Catal.* **2024,** *14* (5), 3287-3297.
14. Sheng, Y.; Xie, J.; Yang, R.; Yu, H.; Deng, K.; Wang, J.; Wang, H.; Wang, L.; Xu, Y., Modulating Hydrogen Adsorption by Unconventional p-d Orbital Hybridization over Porous High-Entropy Alloy Metallene for Efficient Electrosynthesis of Nylon-6 Precursor. *Angew. Chem. Int. Ed.* **2024,** *63* (44), e202410442.

15. Zhang, F.; Fan, Q.-Y.; Huang, Y.-C.; Li, H.; Zou, H.; Li, Y.; Zou, Y.; Wang, S.; Yang, C.; Lu, Y.; Yang, H., A Pickering-emulsion-droplet-integrated electrode for the continuous-flow electrosynthesis of oximes. *Nat. Synth.* **2025,** *4* (4), 479-487.

16. Zhao, J.; Yang, R.; Liu, C.; Zhang, B.; Wu, Y., Ampere-Level Electrosynthesis of ^{15}N-Labeled Oximes by Alloy Synergy Activation and Hydroxylamine Overhydrogenation Inhibition. *Angew. Chem. Int. Ed.* **2025,** *64* (43), e202516528.

17. Jia, S.; Wu, L.; Tan, X.; Feng, J.; Ma, X.; Zhang, L.; Song, X.; Xu, L.; Zhu, Q.; Kang, X.; Sun, X.; Han, B., Synthesis of Hydroxylamine via Ketone-Mediated Nitrate Electroreduction. *J. Am. Chem. Soc.* **2024,** *146* (15), 10934-10942.

18. Zhao, R.; Wang, Y.; Fu, J.; Zhang, F.; Wen, L.; Zhao, Y.; Guan, B.; Han, B.; Liu, Z., Achieving over 90% Faradaic Efficiency in Cyclohexanone Oxime Electrosynthesis Using the Cu-Mo Dual-Site Catalyst. *J. Am. Chem. Soc.* **2024,** *146* (40), 27956-27963.

19. Zhang, C.; Meng, S. L.; Jing, Y. N.; Wang, C.; Zhang, X. L.; Wang, H. X.; Tung, C. H.; Wu, L. Z., Synergistic C-N Coupling for Efficient Cyclohexanone Oxime Synthesis from Ambient Air by Supported Molecular Catalysts. *Angew. Chem. Int. Ed.* **2025,** *64* (26), e202506546.
